# Harnessing Shannon entropy-based descriptors in machine learning models to enhance the prediction accuracy of molecular properties

**DOI:** 10.1186/s13321-023-00712-0

**Published:** 2023-05-21

**Authors:** Rajarshi Guha, Darrell Velegol

**Affiliations:** 1 Intel Corporation, 2501 NE Century Blvd, Hillsboro, OR 97124 USA; 2 Department of Chemical Engineering, Pennsylvania State University, University Park, PA 16802 USA

**Keywords:** SMILES, Shannon entropy, SEF, Deep neural networks, MLP, GNN, kNN, Machine learning

## Abstract

Accurate prediction of molecular properties is essential in the screening and development of drug molecules and other functional materials. Traditionally, property-specific molecular descriptors are used in machine learning models. This in turn requires the identification and development of target or problem-specific descriptors. Additionally, an increase in the prediction accuracy of the model is not always feasible from the standpoint of targeted descriptor usage. We explored the accuracy and generalizability issues using a framework of Shannon entropies, based on SMILES, SMARTS and/or InChiKey strings of respective molecules. Using various public databases of molecules, we showed that the accuracy of the prediction of machine learning models could be significantly enhanced simply by using Shannon entropy-based descriptors evaluated directly from SMILES. Analogous to partial pressures and total pressure of gases in a mixture, we used atom-wise fractional Shannon entropy in combination with total Shannon entropy from respective tokens of the string representation to model the molecule efficiently. The proposed descriptor was competitive in performance with standard descriptors such as Morgan fingerprints and SHED in regression models. Additionally, we found that either a hybrid descriptor set containing the Shannon entropy-based descriptors or an optimized, ensemble architecture of multilayer perceptrons and graph neural networks using the Shannon entropies was synergistic to improve the prediction accuracy. This simple approach of coupling the Shannon entropy framework to other standard descriptors and/or using it in ensemble models could find applications in boosting the performance of molecular property predictions in chemistry and material science.

**Supplementary Information:**

The online version contains supplementary material available at 10.1186/s13321-023-00712-0.

## Introduction

Prediction of the physicochemical properties of molecules is one of the most widely used applications in machine learning and is central to the field of chemistry and material science. The characteristic feature of the molecules or descriptor-based machine learning to predict physicochemical properties faces trade-offs between performance accuracy, interpretability of the results and generalizability to different datasets [1, 2]. Developing novel descriptors could partially address such limitations. However, they suffer from target specificity and therefore, generalizability [3]. Deep heterogeneous ensemble learning, on the other hand, could partially address predictive performance and generalizability issues at the expense of interpretability [4, 5]. Therefore, a combination of novel descriptors along with deep ensemble learning might be able to address the shortcomings more effectively. One of the widely available sources of descriptor design is the representation of the molecule itself. It not only provides structural information but grammatically describes motifs, subtle arrangements, bonds and atomic proximities.

In the last few decades, considerable development took place in representing molecules in various string formats such as SMILES (simplified molecular-input line-entry system), SMARTS (SMILES arbitrary target specification), InChiKey (International Chemical Identifier Key), SYBYL line notation (SLN) [6], SMIRKS (SMIles ReaKtion Specification) [7], SELFIES (Self-referencing embedded strings) [8] etc. Different grammatical or representational aspects were encoded by these strings and therefore, could serve as a source rich in molecular and structural information content for potential use in machine learning applications. Additionally, SMILES representations have several different variants like canonical, isomeric, randomized, DeepSMILES [9] etc. It was demonstrated that depending on the type of SMILES representation, a neural network based on models such as RNNs (Recurrent Neural Networks), could learn and perform more accurately [10]. It implies the possibility of using molecular representations to enhance the accuracy as well as the overall prediction performance of neural network models. Despite some limitations of conventional SMILES strings in representing molecules [11], SMILES-based models could be on par with graph-based models for QSAR-related (Quantitative Structure–Activity Relationship) applications [12]. However, a generalized and simpler approach is required to use string representation in any machine learning model.

Tokenization utilized string representations of molecules efficiently in natural language processing (NLP) models, reduced the dimensionality of embedding space and also helped in the interpretability aspect of the machine learning models by providing attention scores [13]. However, converting molecular representation to vectors using word embedding was only marginally effective or ineffective in terms of machine learning performance, apart from computing bulky matrices and resulting in higher computational costs [14]. Additionally, comparison between different molecules is not straightforward while using tokenized embedding. In this context, the information content of molecules could be represented by Shannon entropies [15, 16] of the tokens referenced to a standard vocabulary of all possible tokens. This information-theoretic approach [16–18] could yield a unique numerical value that is easier to use and compare with other molecules and thereby, could avoid bulky, high-dimensional, computationally expensive matrix processing. In this context, SHED (SHannon Entropy Descriptors) descriptors are relevant which extract the entropy associated with specific features of atom pair distribution over the topology of the molecule [19].

Though topological descriptors are computationally faster to evaluate, the abstractions and interpretations are often not straightforward [20]. On the other hand, entropy-based descriptors estimated from graph-based information indices were computationally complex to evaluate [21]. It was demonstrated that descriptor-based machine learning models could be competitive and execute even faster than graph neural network (GNN) models [22]. Interestingly, it was also shown that simple machine learning models, such as kNN (*k*-nearest neighbor), are competitive and often perform better than GNN models in predicting the QSAR space of various targets of pharmacological importance [23]. Therefore, an efficient descriptor could be engineered to adopt tokenization which has the advantage of interpretability and is expected to be more explainable than traditional topological or information-theoretic descriptors [24]. The descriptor should be simple to define, computationally faster to evaluate and should demonstrate effectiveness across different model types.

Here, we describe a simple framework of Shannon entropies (abbreviated as SEF: Shannon entropy framework) associated with the tokenized and/or character representation of molecules analogous to a molecular descriptor in deep neural networks and in general machine learning models to predict or classify molecular properties more efficiently (Table 1). SEF features are based on estimates of Shannon entropies generated from the tokens and characters relevant to string notations using (i) SMILES tokens (Additional file 1: Table S1a), (ii) SMARTS tokens, (iii) InChiKey strings, (iv) fractional Shannon entropies of atoms which are total Shannon entropy of the respective molecular representation weighted by the frequency of atoms of the molecule (Additional file 1: Table S1b) and (v) frequency of different types of bonds present in the molecule or the associated Shannon entropy of bonds (“Methods”). Fractional Shannon entropy was assumed to be the total Shannon entropy distributed among the constituent atoms analogous to the distribution of total pressure among the component species as partial pressures in a gas mixture. In this case, each atom of the molecule gets an estimate of Shannon entropy. As SEF descriptors of the molecule, either a specific type of previously mentioned entropy or a combination of such entropies could be used to enhance the performance of machine learning models.

**Table 1:** List of datasets used in neural/graph-based models to evaluate SEF

Dataset	Target	Model type	Source/reference
IC_50_ values of binding molecules to tissue factor pathway inhibitor	pCheMBL/MW	Regression	EMBL-EBI
BEI values of binding molecules to the tissue factor pathway inhibitor	BEI/MW	Regression	EMBL-EBI
K_i_ values of binding molecules to coagulation factor 11	pCheMBL/MW	Regression	EMBL-EBI
Toxicity classification as per Ames mutagenicity	Toxicity	Classification	TU-BERLIN
Partition coefficient values of binding molecules to the p53-binding protein Mdm2	logP	Regression	OPEN BABEL/EMBL-EBI

In this study, facile multi-layer perceptron (MLP) based deep neural networks were used to demonstrate the applicability of the concept and then extended to hybrid deep neural networks to test the adaptability and generalizability of the SEF  as a descriptor set. We have also used an ensemble of MLP and graph convolutional neural network (GCN) as hybrid neural network-based models, random forest regression as a traditional machine learning model and kNN regression/classification as baseline models in this article. Comparison of SEF to Morgan fingerprint [25] and SHED descriptors [26] across different datasets and using different machine learning models were also investigated to assess the applicability of the proposed descriptor framework.

## Results

### Shannon entropies based on string notations of molecules are useful descriptors for property predictions

There are a few advantages of using numerical reduction of a molecule in the form of the SEF descriptors such as (i) unique numerical representation of each molecule: it facilitates sensitivity to stereochemistry as well as minimal change in numerical value with structural changes of the molecule (Additional file 1: Table S2), (ii) low correlation to other descriptors: Shannon entropies have lower correlation to other standard descriptors (Additional file 1: Fig. S1). Therefore, a combination of such descriptors could be used in specific applications, (iii) target/problem-specific usage: ease of estimation of different Shannon descriptors allows optimized use of the descriptor set for the target-specific performance of the machine learning model. Overall, descriptors of the Shannon framework satisfy several criteria for successful application in QSAR-type problems [20, 27].

**Fig.1:**
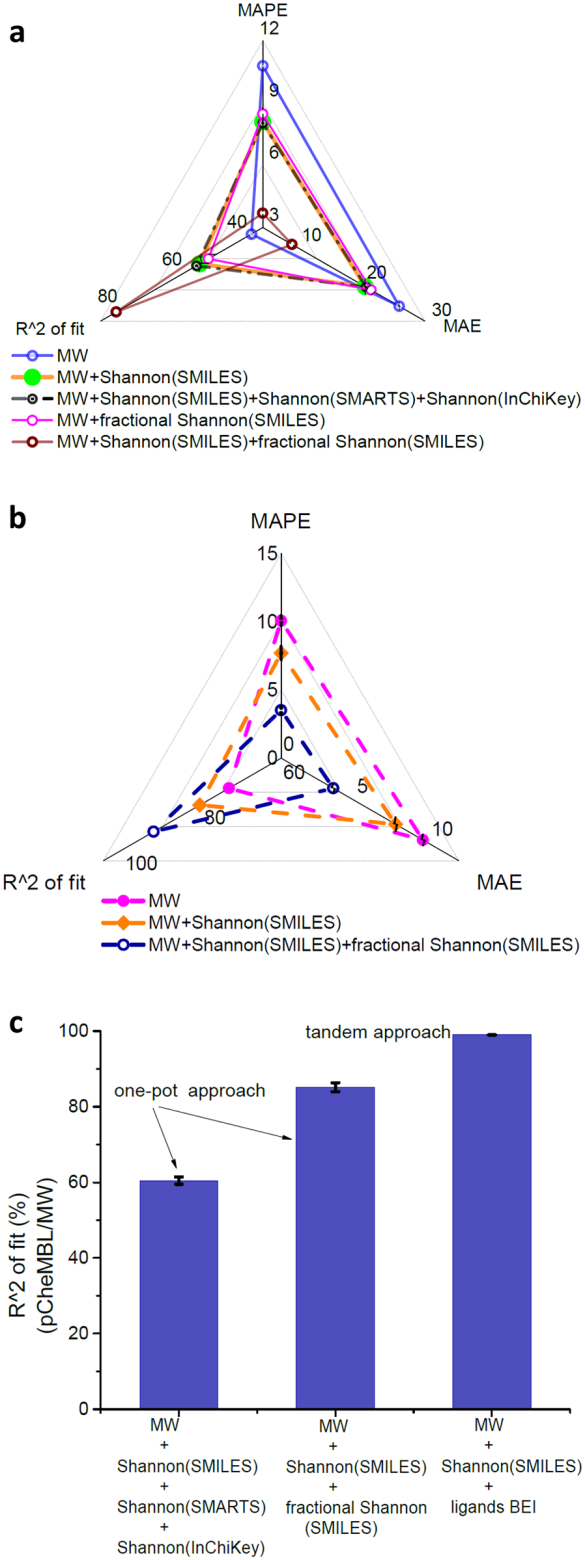
Shannon entropies based on standard tokens and characters derived from a string representation of molecules are efficient descriptors for deep neural network-based property predictions. **a** Comparison of network performance with the addition of different Shannon entropies in the descriptor set. IC_50_ values of tissue factor pathway inhibitor were predicted and analyzed using MAPE, MAE and R^2^ of fit metrics. The descriptor set containing MW, Shannon and fractional Shannon entropies extracted from SMILES showed the best performance in comparison to other descriptors in the triangular radar graph. **b** Cumulative enhancement of network performance using Shannon descriptors depicted in the radar graph. The target was MW normalized BEI of ligands to the tissue factor pathway inhibitor, i.e. in the form of BEI/MW. The SEF set containing MW, Shannon (SMILES) and fractional Shannon (SMILES) showed the best comparative performance in all metrics. **c** Comparison of direct one-pot vs tandem approach to predict IC_50_ values of molecules to the tissue factor pathway inhibitor protein. The tandem approach first predicted BEI as an intermediate step and then predicted IC_50_ values at higher accuracy with the BEI as an input. The model was based on MLP-based deep neural networks and all prediction metrics were averaged over at least 5 independent runs. The scaling factors of metrics were listed in Additional file 1: Table S3

We tested the performance of the deep neural network model for regression-type problems using the following metrics- (i) MAPE (mean absolute percentage error), (ii) R^2^ of fit and (iii) MAE (mean absolute error) or RMSE (root mean squared error) depending on the target. To predict the half-maximal inhibitory concentrations or IC_50_ values of binding molecules (in pCheMBL format) to the protein tissue factor pathway inhibitor (Table 1), we designed a deep neural network primarily composed of MLPs with 4 layers. Among the 3382 data points of this dataset, 2705 data points were used for training and 677 data points were used for validation or testing under the same conditions. We observed an average improvement of 25.5% in MAPE in IC_50_ prediction of binding molecules when a combination of molecular weight (MW), Shannon entropies based on SMILES, SMARTS and InChiKey strings were used compared to only MW as the descriptor (Fig. 1a, Additional file 1: Fig. S2a and Additional file 1: Table S3). However, in this case, the highest comparative improvement in prediction metrics was observed when the Shannon framework was composed of Shannon and fractional Shannon entropies based on SMILES (Fig. 1a). We observed an average improvement of 56.5% in MAPE alone compared to the previous case with Shannon entropies based on SMILES, SMARTS and InChiKey strings (Additional file 1: Fig. S2a and Additional file 1: Table S3). The model performance in this case also excelled kNN-based model (Additional file 1: Table S3 and “Methods”).

Additionally, we used the previous dataset to predict the binding efficiency index (BEI) of ligand molecules (ligands BEI) as the target following the same model. In this case, we observed the best performance while using a combination of MW, Shannon entropy based on SMILES strings (or SMILES Shannon) and fractional Shannon entropies based on total entropy as SMILES Shannon (Fig. 1b, Additional file 1: Fig. S2b and Additional file 1: Table S4). The average improvements in target prediction were 64%, 62% and 25% in MAPE, MAE and R^2^ of fit (%), respectively compared to only MW as a descriptor. It was observed that the SEF descriptor set comprising SMILES Shannon and fractional Shannon, in principle, showed similar or better performance in comparison to other standard molecular descriptors like Morgan fingerprints and also outperformed the kNN-based machine learning model. The prediction of the network was even more accurate when the SEF was used to predict the target BEI than the Morgan fingerprints as descriptors (Additional file 1: Fig. S2c and Additional file 1: Table S4). Following this, we found further enhancement of network performance when both the Shannon framework and Morgan fingerprints were used as molecular descriptors. The synergy between the descriptors caused an average decrease in MAPE ~ 42% compared to the case when only Morgan fingerprints were used. Note that the MW was used as a base descriptor in all the aforementioned cases for comparison purposes only and similar performance was obtained with the SEF descriptors without using the MW (Additional file 1: Table S4).

It is now obvious that the prediction of IC_50_ of the molecules of the dataset could be carried out in a step-by-step sequential manner- (i) first predicting ligands BEI with high efficiency using SEF, and (ii) then using the ligands BEI as a descriptor along with the SEF to finally predict IC_50_ of ligand molecules (Fig. 1c and Additional file 1: Table S5) with higher accuracy. The optimized SEF features consisted of SMILES Shannon and fractional Shannon entropies of atoms based on SMILES. This tandem approach improved the overall prediction accuracy when compared to several one-pot type methods which predicted the IC_50_ of molecules directly using the MW and the corresponding SEF features. The R^2^ of fit (%) was 99% for the tandem case.

### Shannon entropies boost performance as descriptors in both regression and classification-type machine learning models

**Fig. 2:**
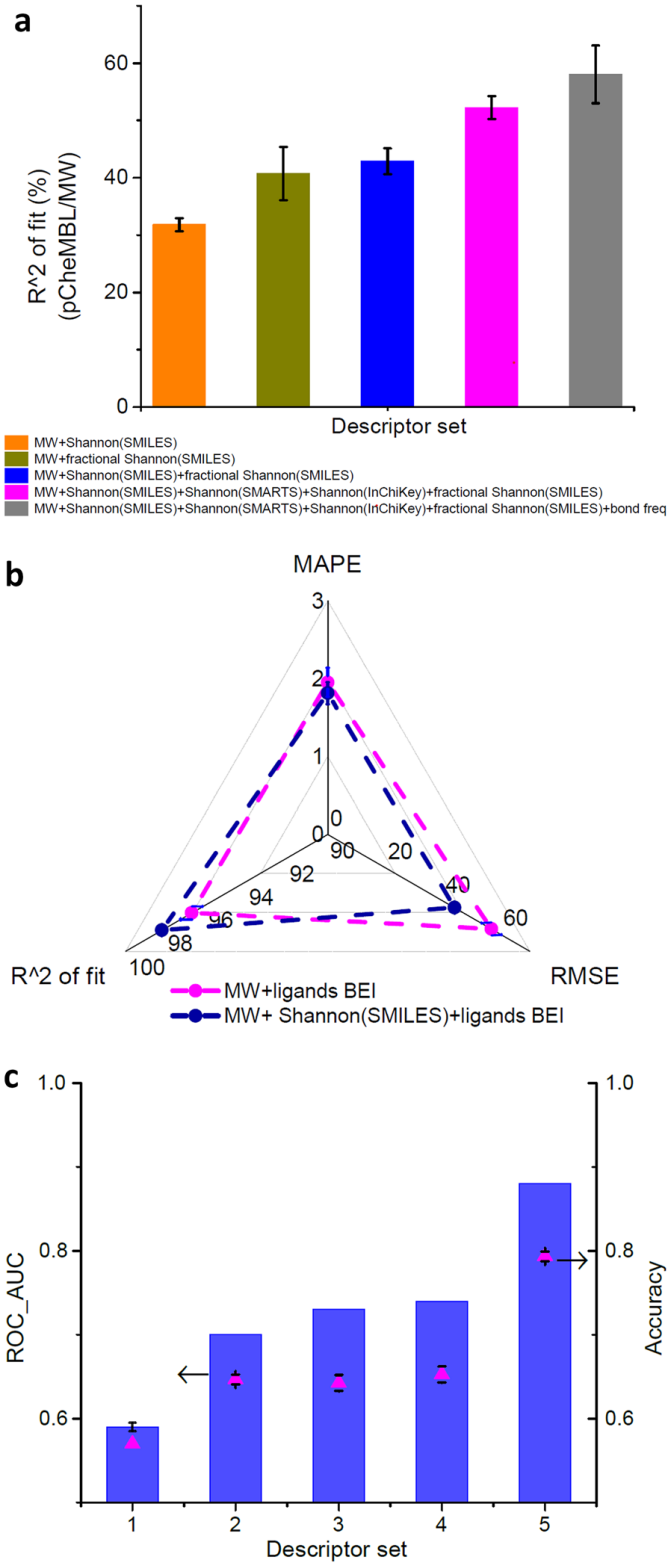
Cumulative performance boost of either regression or classification type problems was attained using the SEF descriptors. **a** Comparison of network performance with cumulative addition of different Shannon entropies in the descriptor set. Ki values of binding molecules to the human coagulation factor 11 were analyzed using the metric R^2^ of fit (%). **b** The addition of the Shannon (SMILES) entropy to the descriptor set consisting of MW and BEI of ligands (ligands BEI) improved the overall performance of the deep neural network. The scaling factors of metrics were listed in Additional file 1: Table S6. **c** The cumulative increase in ROC_AUC and accuracy of the toxicity classification of Ames mutagenicity dataset by cumulative addition of different Shannon entropy-based descriptors. The used descriptor sets were 1. Shannon (SMILES), 2. fractional Shannon (SMILES), 3. fractional Shannon (InChiKey), 4. Shannon (SMILES) + Shannon (SMARTS) + Shannon (InChiKey) + fractional Shannon (InChiKey) + bond freq, and 5. Other descriptors + Shannon (SMILES) + fractional Shannon (SMILES). The other descriptors were listed in Additional file 1: Table S8. All prediction metrics were averaged over at least 5 independent runs

To assess the applicability of the Shannon entropy approach to other datasets, we tested the prediction of inhibition constants or K_i_ values (pCheMBL format) of ligand molecules that could bind to the protein human coagulation factor 11 (or F11). The dataset contained 618 data points among which 525 values were used for training and 93 values were used for testing. Our objective was to achieve cumulative improvement in network performance by using several SEF features as descriptors, including bond frequency information. Bond frequency refers to the occurrence of types of bonds of the molecule as frequency estimates (Additional file 1: Table S6 and “Methods”). On average, the R^2^ of fit (%) was improved by 82.4% when the combination of MW, Shannon entropies based on SMILES, SMARTS, InChiKey, fractional Shannon and bond frequency information were used as descriptors compared to only MW and Shannon entropy based on SMILES (Fig. 2a and Additional file 1: Table S6). The use of the bond frequency feature also enabled the SEF to rival the performance of the kNN-based machine learning model. This observation also demonstrates the importance of frequency information as a molecular descriptor.

Efficiency improvement of the prediction of the network with Shannon entropy was also achieved when ‘ligands BEI’ was used as one of the descriptors (Fig. 2b). To predict K_i_ values of the dataset mentioned above, BEI is arguably one of the most useful features. When Shannon entropy based on SMILES was used in conjunction with MW and BEI of ligands in the descriptor set, network performance was improved in all metrics (Fig. 2b and Additional file 1: Table S7). We tested this trained model with the anticoagulant drug Milvexian which has a reported K_i_ of 0.11 nM [28]. This data was not present in the training dataset. Upon querying our model with Milvexian as input, the prediction of K_i_ of Milvexian was ~ 0.15 nM which was close to the reported value.

The Ames mutagenicity dataset was used to assess the performance of the SEF descriptors in classification-type models [29]. The dataset contained 6506 usable data points which were divided into 5530 training and 976 testing data. Here also our objective was to observe cumulative improvements in two metrics—(i) ROC_AUC (area under the curve for receiver operating characteristic) and (ii) accuracy by using different Shannon entropies as descriptors. The average performance increase was the highest in both the metrics when a combination of Shannon and fractional Shannon entropies were used as features of the descriptor (Fig. 2c, case 4) compared to only Shannon entropy based on SMILES (Fig. 2c, case 1). In this case, the fractional Shannon entropies of atoms were evaluated from Shannon entropy based on the InChiKey string. However, a kNN-based classification model outperformed the MLP-based classification model using only the SEF descriptors (Additional file 1: Table S8). Therefore, a set of other descriptors were evaluated which could work in combination with SEF to result in better performance than the kNN-based model (Additional file 1: Table S8). Improvement of model performance (ROC_AUC ~ 0.88 and accuracy ~ 0.8) was achieved when the combined descriptors (Additional file 1: Table S8), estimated using the rdkit package and MHFP encoder [30], were used along with the SEF descriptor set.

We have also assessed the performance of a hybrid network using a combination of MLP and CNN models on both the pCheMBL F11 and Ames mutagenicity datasets. The MLP part of the network was trained with the discussed SEF features for respective datasets, i.e. a combination of Shannon entropies based on SMILES, SMARTS, InChiKey strings and fractional Shannon entropies based on SMILES in the case of the former dataset (Additional file 1: Table S9). In the MLP part of the network of the later dataset, we used the descriptors mentioned previously (Additional file 1: Table S8). The CNN part of the network was trained on two-dimensional images of molecules constructed from their respective SMILES strings in the case of both datasets. We found comparable performance between models with only MLP-based deep neural networks and hybrid MLP and CNN-based deep neural networks for both datasets (Additional file 1: Fig. S3a, b), implying that no synergy and performance gain upon using hybrid MLP and CNN-based models.

### MLP and GNN ensemble models using Shannon entropies are synergistic in enhancing the prediction accuracy of molecular properties

**Fig. 3:**
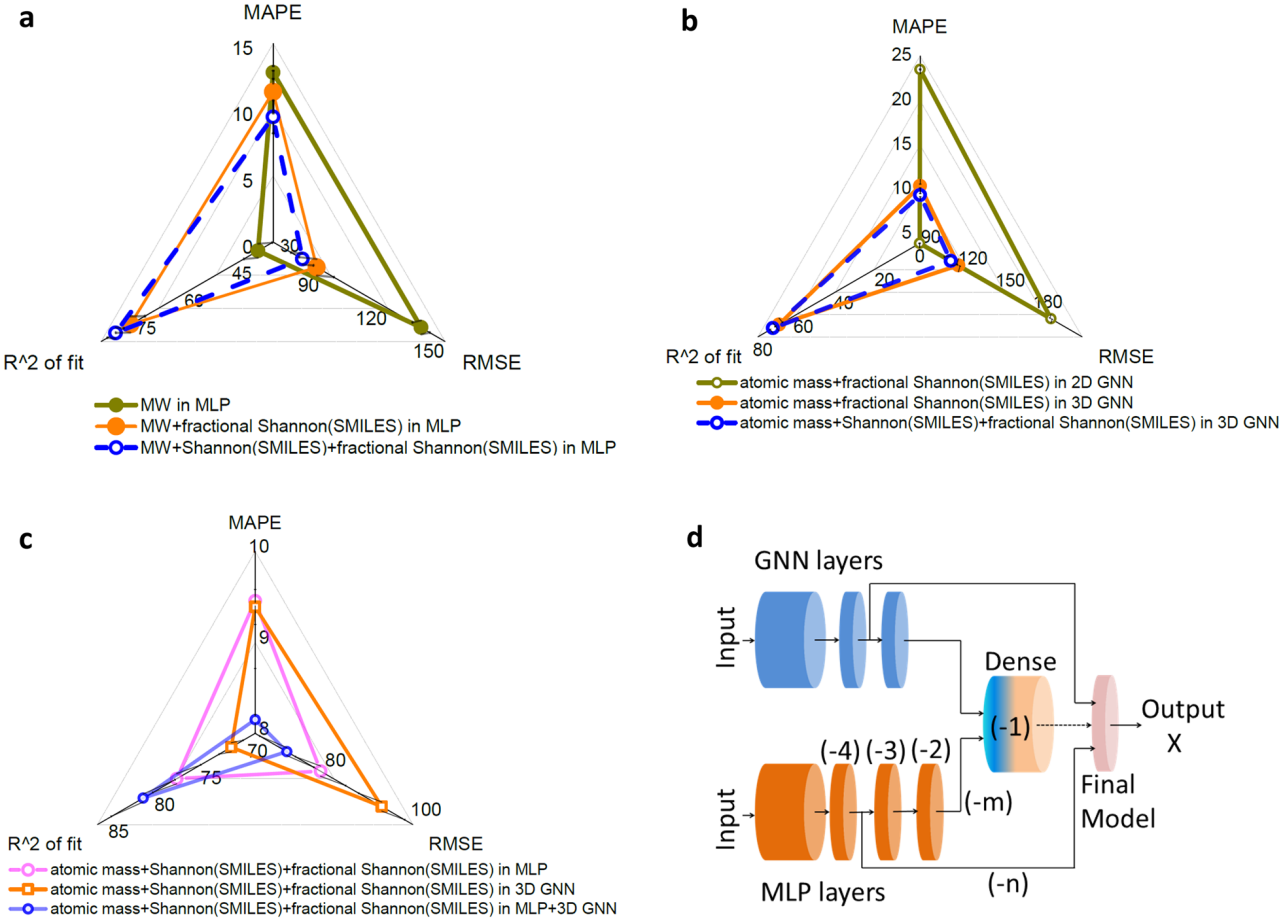
Ensemble models of MLP and GNN architecture-based deep neural networks using the SEF descriptors to increase the prediction accuracy of molecular properties. **a** Comparison of model performance of MLP-based deep neural network with cumulative addition of different Shannon entropies to the descriptor set. Predictions of partition coefficient (logP) values of binding molecules to the p53-binding protein Mdm2 were analyzed in the triangular radar plot. A combination of MW, and Shannon entropies based on SMILES Shannon and fractional Shannon (SMILES) showed the best comparative performance (blue dash). **b** The 3-dimensional (3D) GNN (GCN-based) model performed better than the 2-dimensional (2D) GNN (GCN-based) model under the same training and testing conditions. When SMILES Shannon was used as an additional node feature, the performance of 3D GNN improved further. **c** The hybrid model of MLP and 3D GNN architectures performed better than the individual MLP or 3D GNN-based model with the same set of Shannon entropy-based node features. The relevant connection was (− 2, − 4) from MLP layers. **d** Schematic of the MLP-GNN hybrid network architecture which used the (− m, − n) connections from MLP layers to the dense and final model, respectively. The scaling factors of all metrics were listed in Additional file 1: Table S10 and all prediction metrics were averaged over at least 5 independent runs

To further generalize the applicability of the Shannon entropy approach, we used another dataset consisting of partition coefficient (logP) values of ligand molecules that could bind to the p53-binding protein Mdm2. The dataset contained 440 data points out of which 374 training and 66 testing splits were performed to assess the usefulness of SEF using the MLP-based model. Significant performance improvement resulted while using fractional Shannon entropy based on SMILES (Fig. 3a and Additional file 1: Table S10). For example, R^2^ of fit (%) increased from 33.72 ± 4.43 to 73.78 ± 4.58 simply by incorporating fractional Shannon entropy in the descriptor set. Further improvement in performance metrics was achieved by incorporating Shannon entropy based on SMILES into the previous descriptor set (Fig. 3a).

Extending the Shannon framework to GNNs, we first used a simplified GNN model composed of nodes representing the atomic mass and the fractional Shannon entropy (SMILES-based) of atoms of a molecule. An array of elements from the periodic table covering the dataset was used as an input to estimate the frequency of occurrence and fractional Shannon entropy of that particular atom (“Methods”). The edge features were simply bond connectivity and edge weights were normalized bond order. We defined this model as 2D (2-dimensional) GNN as no 3D (3-dimensional) or conformational information was used in the node features of graphs. We used a GCN network using the StellarGraph package [31] to implement the GNN model.

We compared the results of the 2D GNN model to the 3D GNN model where 3D information of the local density of atoms based on pairwise distance was used corresponding to the lowest energy conformer (Additional file 1: Fig. S4). This topological descriptor was used along with the atomic mass and fractional Shannon entropies (SMILES-based) as node features. As expected, there was significant performance improvement with the 3D GNN as compared to the 2D GNN model (Fig. 3b). Further improvement in the performance of the 3D GNN model, for example, ~ 10% improvement in MAPE was achieved when Shannon entropy based on the SMILES was used as a node feature along with the previous features of the descriptor set (Fig. 3b).

Unlike the case of hybrid MLP and CNN-based models, we observed performance improvement from a synergy between MLP and 3D GNN-based deep neural network models. The hybrid of MLP and 3D GNN architecture was able to perform better than either MLP or 3D GNN model under the same training and testing conditions (Fig. 3c). This was partly because the output of the individual network i.e. outputs of the MLP and GNN models were ensembled and further passed through a deep neural network to train the dataset more efficiently. Similar performance enhancement was also observed for models containing hybrid MLP and 2D GNN architectures (Additional file 1: Fig. S5a).

We further investigated the dependency of model performance on network connections between MLP and GNN architectures. We define the final model as part of the hybrid model after the final dense layers (Fig. 3d). While the output from the GNN layers to the final model was kept constant, we skipped a few final layers from the MLP network to connect to the final model and observed that the overall performance of property prediction was improved (Fig. 3d). For example, connecting (− 2, − 4) layers of the MLP branch to the final, hybrid model was found to be more accurately predictive than connecting (− 2, − 3) layers to the final model (Additional file 1: Fig. S5b). Similarly, connecting (− 2, − 3) layers was more accurately predictive than connecting (− 2, − 2) layers (Additional file 1: Table S10). Here, ‘− 1’ refers to the final dense layers, ‘− 2’ refers to the layer before the dense and so on. The format of notation used, for example, (− m, − n) refers to the layer numbers of the MLP network connected to the dense layer and the final model, respectively. Specifically, ‘− m’ refers to the output of the MLP layer to the dense layer for estimating the output to fit the final model and ‘− n’ refers to the output of the MLP layer as one of the inputs to the final model (Fig. 3d and Additional file 1: Fig. S6). The other input to the final model was the output of the penultimate layer of the GNN network which was kept constant as mentioned earlier. This strategy of tweaking the inputs to the final, hybrid model from one of its constituent networks (MLP for example) to enhance the overall prediction performance was applied to architectures using both 2D and 3D GNN with consistent results (Additional file 1: Table S10).

However, it is to be noted that an optimized kNN model could rival the performance of combined MLP and GNN models in predicting the logP values. We found that only ensemble architectures of 3D GNN, where (− 2, − 4) layers of MLP were connected, could excel in the performance demonstrated by the kNN model (Additional file 1: Table S10). Ensemble architecture of 3D GNN and MLP-based neural networks using the SEF descriptors also outperformed the kNN model when a different dataset (CHEMBL4691) and target values (pCheMBL) were used (Additional file 1: Table S10).

### SEF descriptors across different tested datasets for regression models rivaled Morgan fingerprints and SHED descriptors in performance

To demonstrate the applicability of SEF descriptors across different datasets and models, we tested them on several datasets using (a) deep neural network architecture comparing Morgan, SEF and SHED descriptors (Table 2) and (b) random forest ensemble architecture comparing Morgan, SEF, SHED, a hybrid of Morgan and SEF and hybrid of SHED and SEF descriptors (Table 3). The kNN baseline for each dataset is also provided as a comparison. Table 2 lists the MAE values from the kNN baseline models and also the comparison mentioned in (a). We primarily used Shannon entropy and fractional Shannon entropies based on SMILES representation as features of SEF in the deep neural network models. As noted by Janela and Bajorath [23], we also found that a simple kNN model could rival deep neural architecture-based models using different descriptors (Table 2 and Additional file 1: Table S11). However, among neural network-based architectures, SEF descriptor-based models performed better across the different datasets tested (Additional file 1: Table S11).

**Table 2:** List of the used datasets and comparison of descriptors in MLP-based deep neural network models

Dataset (Target ID)	Target variable	Sample size	kNN (MAE)	Morgan (MAE)	SEF (MAE)	SHED(MAE)	Source/reference
CHEMBL 3713062^a^	BEI	3382	6.47	5.00 ± 0.13	3.70 ± 0.15	10.74 ± 0.48	EMBL-EBI
CHEMBL 204	BEI	1777	4.20	5.03 ± 0.20	4.23 ± 0.05	10.41 ± 0.30	EMBL-EBI
CHEMBL 2842	BEI	4164	4.90	4.50 ± 0.14	4.07 ± 0.08	9.76 ± 0.24	EMBL-EBI
CHEMBL 274	BEI	1950	2.64	3.54 ± 0.24	2.90 ± 0.05	4.95 ± 0.02	EMBL-EBI
CHEMBL 3974	BEI	725	3.80	5.00 ± 0.35	3.52 ± 0.11	9.23 ± 0.03	EMBL-EBI
CHEMBL 2820	BEI	663	2.70	3.33 ± 0.25	2.92 ± 0.11	4.58 ± 0.13	EMBL-EBI
CHEMBL 2815	BEI	3182	3.90	4.21 ± 0.21	3.84 ± 0.07	7.25 ± 0.02	EMBL-EBI
CHEMBL 4691	pCheMBL	859	2.25	2.14 ± 0.08	1.94 ± 0.03	2.70 ± 0.02	EMBL-EBI

^a^The scaling factor of MAE was 10^5^ and for the rest of the Target IDs the scaling factor was 10^3^

**Table 3:** Comparison of Morgan, SEF, SHED and hybrid descriptors in random forest regression-based models

Dataset (Target ID)	Target variable	Morgan (MAE)	SEF (MAE)	SHED (MAE)	SEF + Morgan (MAE)	SEF + SHED (MAE)
CHEMBL 3713062^a^	BEI	4.75 ± 0.01	2.92 ± 0.03	4.51 ± 0.02	2.36 ± 0.02	2.92 ± 0.03
CHEMBL 204	BEI	4.60 ± 0.05	3.65 ± 0.03	7.10 ± 0.05	3.23 ± 0.00	3.60 ± 0.00
CHEMBL 2842	BEI	4.10 ± 0.02	3.61 ± 0.00	8.35 ± 0.05	3.13 ± 0.01	3.60 ± 0.01
CHEMBL 274	BEI	2.20 ± 0.04	2.10 ± 0.01	3.85 ± 0.01	1.85 ± 0.00	2.10 ± 0.00
CHEMBL 5023^b^	logP	0.51 ± 0.00	0.43 ± 0.01	0.63 ± 0.01	0.43 ± 0.00	0.40 ± 0.00

(i) ^a^The scaling factor of MAE was 10^5^, ^b^the scaling factor was 1 and for the rest of the Target IDs the scaling factor was 10^3^ and (ii) ^b^MAE with the kNN-based model was 0.50

Similar results were obtained when random forest regression models were used to compare different descriptors mentioned in (b). We noticed two interesting aspects of SEF descriptors comparing across different datasets using the random forest model- (i) other descriptor-based models (Morgan and SHED) could perform better when used in combination with SEF descriptors (Additional file 1: Table 3 and Additional file 1: Table S12) and (ii) SEF descriptor-based models were computationally faster under the same conditions across the tested datasets, except for the case of the target CHEMBL3713062. In this case, the average molecular weight of binding molecules was higher compared to other datasets. We used an optimized set of features in constructing the SEF descriptors depending on the specific dataset used in random forest regression models (Additional file 1: Table S13). In the context of the used datasets, SEF-based random forest ensemble models outperformed all other descriptor-based models and kNN models, in comparison, as well (Additional file 1: Table S12).

We also investigated the performance of the SEF descriptors in comparison to other descriptors by random shuffling of target values of the dataset for a few cases (Additional file 1: Table S14) using the random forest models. We observed quite similar results when such randomized structure-target space was used as compared to the original space. The standard deviation values were higher in randomized cases as compared to the original.

## Conclusions

We have described a method to use material information content to enhance the performance of deep neural networks and general machine learning models. We tapped the Shannon entropies associated with material representation in various string formats and used them as molecular descriptors, defined as SEF, to increase the prediction accuracy of deep neural networks, hybrid neural architectures, and also general machine learning models. Total Shannon entropy of molecules, atom-wise fractional Shannon entropies and frequency information of molecular structures such as type of bonds or Shannon entropy of bonds were effective as descriptors in machine learning applications. These descriptors had a relatively lower correlation to other standard descriptors, were sensitive to the stereochemistry and caused a lower change in values in response to smaller structural variations. Additionally, SEF descriptors were found to be competitive with standard descriptors such as Morgan fingerprints and SHED as well as kNN-based machine learning models in the QSAR space [23]. Various datasets and target types encompassing molecules of pharmaceutical significance were used to demonstrate the applicability of the SEF descriptors in QSAR modeling. We observed performance enhancement in both regression and classification-type problems when the SEF descriptors were used in the defined representations. Interestingly, we found synergy between MLP and GNN architectures using SEF and the resulting hybrid networks performed more accurately than their constituent counterparts. SEF also demonstrated synergy with other descriptors, which could further boost the performance of machine learning models. As features of SEF could readily reduce the material information to simple, distinct numerical representation, it could potentially be used in material informatics, screening, design and optimization.

## Methods

### SEF descriptors

1. Generate tokens from atom-wise or SMILES pair encoding tokenizer [32]. For InChiKey strings, use a character-wise tokenizer to extract the tokens. The generated token set is: ${x}_{i}\in X$
2. Estimate the frequency of the token ${x}_{i}$ as: ${f}_{{x}_{i}}= n/N$. Where $n$ is the number of occurrences of a token ${x}_{i}$ and $N$ is the total occurrence of all the tokens. The number of unique tokens generated is $k$.
3. The estimated Shannon entropy of the m-th molecule (${S}_{m}$) of the dataset is based on the token frequencies:${S}_{m}= -\sum_{i}^{k}{f}_{{x}_{i}}{log}_{2}{f}_{{x}_{i}}$ (Additional file 1: Table S1a). Note that ${S}_{m}$ could be evaluated not only from SMILES representation but from any other structural or scientific notations, for example, SELFIES [8], BigSMILES [33] etc.
4. For estimating fractional Shannon entropy, the following steps were followed:
  - Define the set of atoms present in the dataset,
  - Construct a dictionary of atoms and their occurrence in the molecule,
  - Estimate the Shannon entropy based on steps 1–3 above,
  - The fractional Shannon entropy (${s}_{{a}_{j}}$) of the atom ${a}_{j}$ within a molecule is calculated analogously to the partial pressure of a component in a gas mixture: ${s}_{{a}_{j}}= {f}_{{a}_{j}}{S}_{m}$. Where ${f}_{{a}_{j}}$ is the ratio of the number of atoms ${a}_{j}$ of a species to the total number of atoms within the molecule of the dataset. For the same atomic species within a molecule, all atoms have the same fractional Shannon entropy similar to the partial pressure of a component in a gas mixture.
  - Since fractional Shannon entropy represents atom-wise entropy, appropriate padding is required to account for the varying number of atoms per compound of the dataset. For example, if a dataset has N as the maximum number of atoms in a molecule, the array of fractional Shannon entropy of any molecule with m atoms would have padding (N-m)/2 on each side (Additional file 1: Table S1b). We used padding with zeros.
5. For estimation of bond frequency and Shannon entropy of bonds, predefine a list covering different types of bonds in the dataset, for example:$${b}_{type}$$ = ['SINGLE', 'DOUBLE', 'TRIPLE', 'QUADRUPLE', 'AROMATIC', 'HYDROGEN', 'IONIC']. Then the bond frequency could be estimated as an array where each element is the number of occurrences of the bond type to the total number of pre-defined bonds present in the compound. A reduction of the bond frequency ($${f}_{{b}_{i}}$$) to the Shannon entropy of bonds ($${S}_{bf}$$) could also be used: $${S}_{bf}= -\sum_{i}^{l}{f}_{{b}_{i}}{log}_{2}{f}_{{b}_{i}}$$, where ‘$$l$$’ is the number of unique bond types ($${b}_{type})$$.

Evaluation of kNN baseline: the *k*-nearest neighbor model was developed by considering *k* average property values based on a similarity metric between the test sample and the training dataset. The similarity metric was estimated based on the Tanimoto similarity and was calculated using an extended connectivity fingerprint with diameter 4 (ECFP4) utilizing the RDKit package. For performance optimization, a sweep over *k* = 1, 2 and 3 was carried out and the best possible model was chosen.

Random forest ensemble models: an ensemble of decision trees was used as a regression model using the scikit-learn package. The grid search method of optimization was used over the following search parameters: (i) the number of decision trees: [25, 100, 200], (ii) the minimum number of samples per leaf node: [1,2,5] and (iii) the minimum number of samples during split: [2,3,5]. A minimum of 5 cross-validations were used for each model.

Evaluation of SHED descriptors: unfolded version of SHED descriptors was generated using a custom script using jCMapper and utilizing the executable java file ‘jCMapperCLI.jar’ [26]. The SHED descriptor data for each dataset was saved in.csv file format and later used in different machine learning models, separately or in combination with other descriptors.

Refer to the shared repository https://github.com/ShannonDescriptors?tab=repositories and follow the below links in the Availability of data and materials section for implementation and examples.

## Supplementary Information

**Additional file 1.** Figures and Tables:** Fig.S1.** Lower correlation of Shannon entropy (SMILES) to other standard descriptors.** Fig.S2.** Shannon entropies based on standard tokens derived from string representations (SMILES, SMARTS, INCHIKEY etc.) of molecules are efficient descriptors for deep neural network-based property predictions.** Fig.S3.** A hybrid neural network combining MLP and CNN models shows comparable prediction performance to only MLP-based models in both classification and regression problems.** Fig.S4.** Schematic with a stepwise depiction of the used algorithm of the hybrid MLP + 3D GNN-based model to predict the logP values of binding molecules to the p53-binding protein Mdm2.** Fig.S5.** Deep neural network model with MLP and 2D GNN architectures in an ensemble performs better than only the 2D GNN based model and the prediction accuracy depends on the connections from MLP layers to the final, hybrid model.** Fig.S6.** Schematic presentations of (-2,-4) and (-2,-3) connections from the last few MLP layers to the final model of the hybrid MLP + 2D/ 3D GNN-based deep neural network.** Table S1a.** An example of numerical reduction of a molecule in the form of Shannon entropy.** Table S1b.** An example of numerical reduction of a molecule in the form of fractional Shannon entropy.** Table S2.** Stereochemistry-sensitive numerical reduction of molecules in the form of Shannon entropies by using a combination of SMILES and InChiKey strings.** Table S3.** Network performance metrics for prediction of IC_50_ values of binding molecules to tissue factor pathway inhibitor (target: pCheMBL/MW, MLP-based deep neural model).** Table S4.** Network performance metrics for prediction of BEI values of binding molecules to the tissue factor pathway inhibitor (target: BEI /MW, MLP-based deep neural model).** Table S5.** Network performance metrics for prediction of IC_50_ values of binding molecules to tissue factor pathway inhibitor in tandem approach (target: pCheMBL/MW, MLP-based deep neural model).** Table S6.** Network performance metrics for prediction of K_i_ values of binding molecules to coagulation factor 11 (target: pCheMBL/MW, MLP-based deep neural model).** Table S7.** Network performance metrics for prediction of K_i_ values of binding molecules to coagulation factor 11 (target: pCheMBL/MW, MLP-based deep neural models).** Table S8.** Network performance metrics for toxicity classification as per Ames mutagenicity (target: toxicity binary classification, MLP-based & CNN&MLP –based deep neural models).** Table S9.** Network performance metrics for prediction of K_i_ values of binding molecules to coagulation factor 11 (target: pCheMBL/MW, MLP-based and CNN&MLP –based deep neural models).** Table S10.** Network performance metrics for prediction of partition coefficient (logP) values of binding molecules to the p53-binding protein Mdm2 (target: logP) and IC_50_ (pCheMBL) values of target ID CHEMBL4691.** Table S11.** Network performance metrics for prediction of BEI and pChEMBL values across different target datasets using MLP-based deep neural network architecture.** Table S12.** Comparison of Morgan, SEF, SHED, SEF+Morgan and SEF+SHED descriptors in random forest regression-based models.** Table S13.** Features used in constructing SEF descriptors for optimum performance in random forest regression-based models.** Table S14.** Comparison of Morgan, SEF and SHED descriptors in random forest regression-based models with completely random y-label targets.

## Data Availability

The scripts, data and usage directions are available online at the following GitHub repositories: (1) IC_50_ and BEI models: https://github.com/ShannonDescriptors/MLP-based-DNN-models-with-Shannon-entropy-framework. (2) K_i_ models: https://github.com/ShannonDescriptors/MLP-and-hybrid-MLP-CNN-models-with-Shannon-entropy-framework. (3) Ames mutagenicity models: https://github.com/ShannonDescriptors/MLP-CNN-and-hybrid-MLP-CNN-models-for-Ames-mutagenicity-with-Shannon-entropy-framework. (4) logP models: https://github.com/ShannonDescriptors/MLP-GNN-and-hybrid-MLP-GNN-models-with-Shannon-entropy-framework. (5) Comparison of descriptors: https://github.com/ShannonDescriptors/ComparisonBeweenDescriptors. Please refer to the ‘README’ section of each repository for directions to use. Scripts to estimate SEF descriptors and all the associated models are readily available to use.
