# Supplementary material for: Harnessing Shannon entropy-based descriptors in machine learning models to enhance the prediction accuracy of molecular properties
Source: J Cheminform. 2023 May 21;15:54. doi: 10.1186/s13321-023-00712-0 (PMC10200055; doi:10.1186/s13321-023-00712-0)
Supplement: Supplementary file 1 — Additional file 1. Figures and Tables: Fig.S1. Lower correlation of Shannon entropy (SMILES) to other standard descriptors. Fig.S2. Shannon entropies based on standard tokens derived from string representations (SMILES, SMARTS, INCHIKEY etc.) of molecules are efficient descriptors for deep neural network-based property predictions. Fig.S3. A hybrid neural network combining MLP and CNN models shows comparable prediction performance to only MLP-based models in both classification and regression problems. Fig.S4. Schematic with a stepwise depiction of the used algorithm of the hybrid MLP + 3D GNN-based model to predict the logP values of binding molecules to the p53-binding protein Mdm2. Fig.S5. Deep neural network model with MLP and 2D GNN architectures in an ensemble performs better than only the 2D GNN based model and the prediction accuracy depends on the connections from MLP layers to the final, hybrid model. Fig.S6. Schematic presentations of (-2,-4) and (-2,-3) connections from the last few MLP layers to the final model of the hybrid MLP + 2D/ 3D GNN-based deep neural network. Table S1a. An example of numerical reduction of a molecule in the form of Shannon entropy. Table S1b. An example of numerical reduction of a molecule in the form of fractional Shannon entropy. Table S2. Stereochemistry-sensitive numerical reduction of molecules in the form of Shannon entropies by using a combination of SMILES and InChiKey strings. Table S3. Network performance metrics for prediction of IC50 values of binding molecules to tissue factor pathway inhibitor (target: pCheMBL/MW, MLP-based deep neural model). Table S4. Network performance metrics for prediction of BEI values of binding molecules to the tissue factor pathway inhibitor (target: BEI /MW, MLP-based deep neural model). Table S5. Network performance metrics for prediction of IC50 values of binding molecules to tissue factor pathway inhibitor in tandem approach (target: pCheMBL/MW, MLP-based deep neural model). Table S6 [file 13321_2023_712_MOESM1_ESM.docx]

**Harnessing Shannon entropy-based descriptors in machine learning models to enhance the prediction accuracy of molecular properties**

^a^Rajarshi Guha and ^b^Darrell Velegol

^a^Intel corporation, 2501 NE Century Blvd, Hillsboro, OR 97124

^b^Department of Chemical Engineering, Pennsylvania State University,

University Park, Pennsylvania, 16802, USA

*Correspondence should be addressed to R.G. ([rajarshiche@gmail.com](mailto:rajarshiche@gmail.com))

Supplementary Information

| 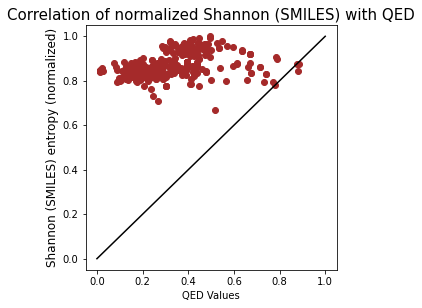  **a** |
| --- |
| 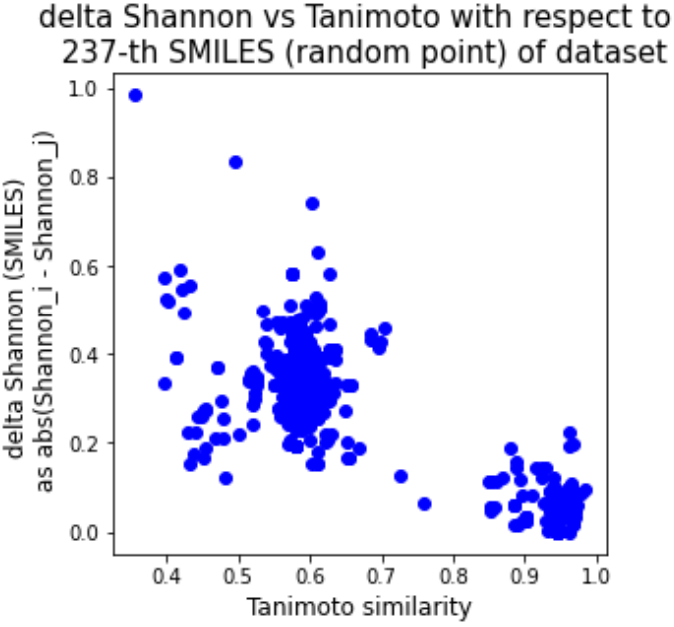  **b** |

**Fig.S1. Lower correlation of Shannon entropy (SMILES) to other standard descriptors.** The used dataset was logP values of binding molecules to the p53-binding protein Mdm2. (a) Low correlation of Shannon entropies (SMILES) normalized with maximum Shannon entropy of the dataset to the QED descriptor estimated using rdkit package. R^2^ of fit (%) = 0.16. (b) Correlation between difference or delta (absolute) in Shannon (SMILES) entropy between a molecule (i-th) of the dataset and randomly selected 237^th^ molecule (j-th) of the dataset and the Tanimoto similarity between those molecules estimated using rdkit package. R^2^ of fit (%) = 0.59.

| **a**  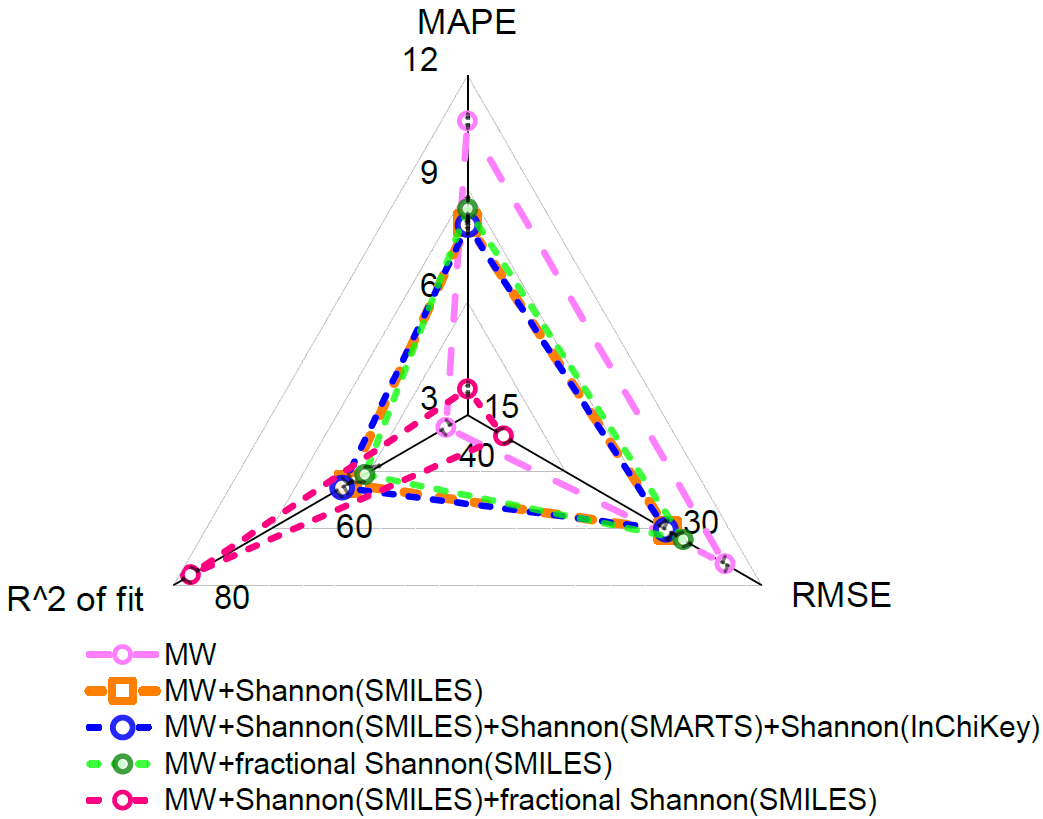 |
| --- |
| 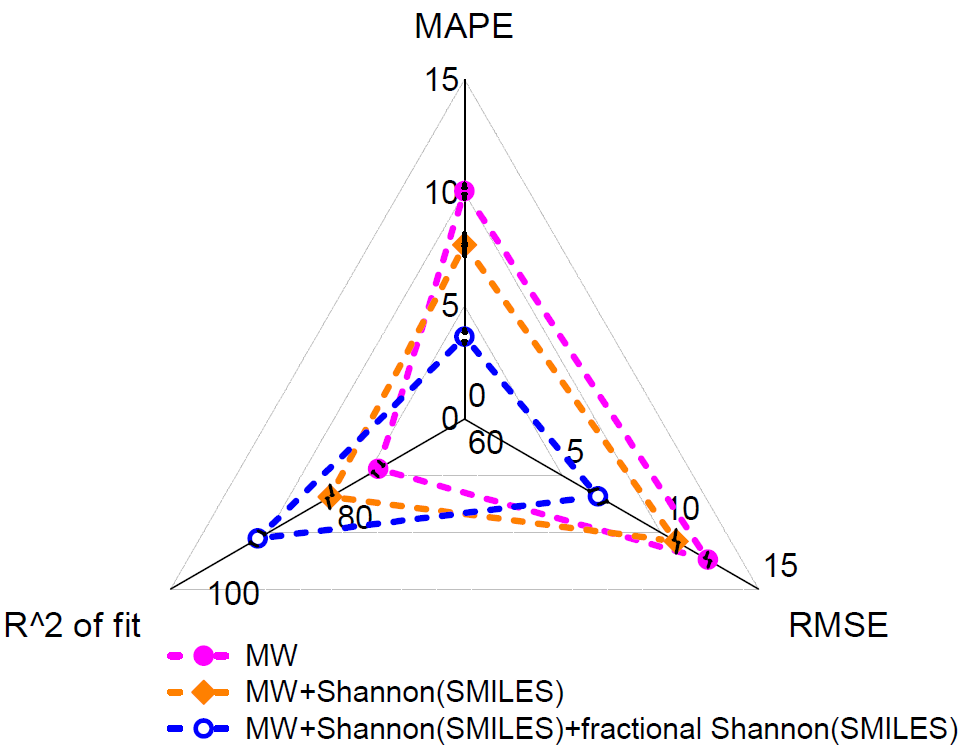  **b** |
| 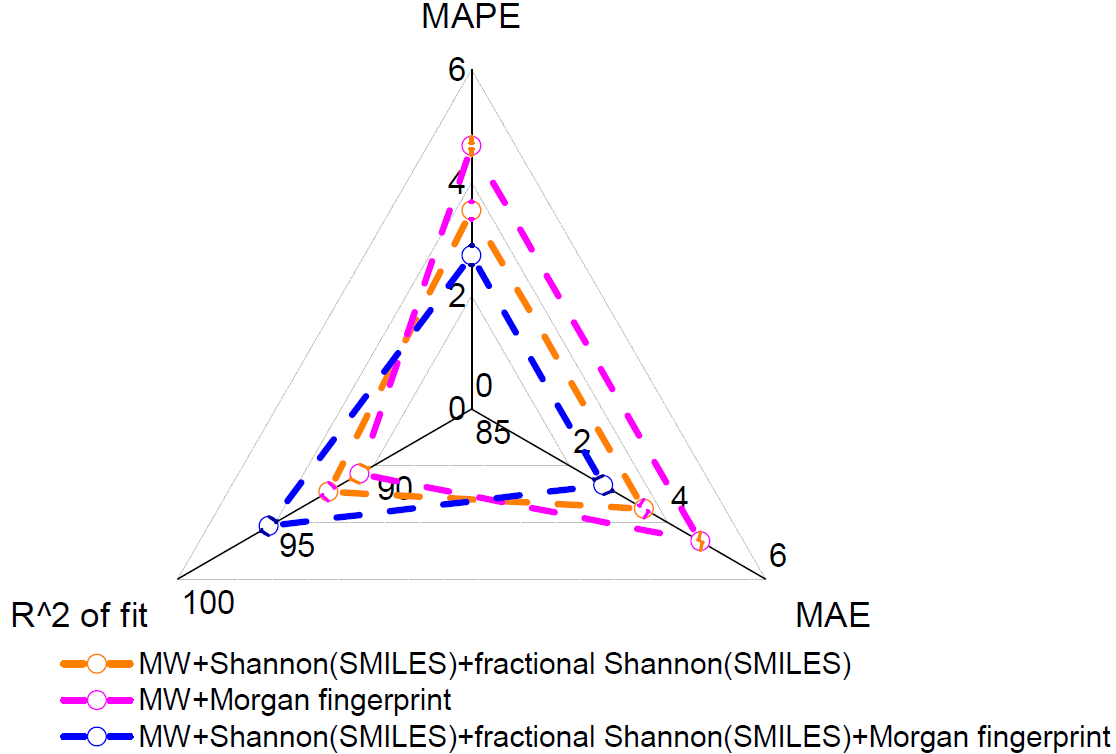  **c** |

**Fig.S2. Shannon entropies based on standard tokens derived from string representations (SMILES, SMARTS, INCHIKEY etc.) of molecules are efficient descriptors for deep neural network-based property predictions.** (a) Comparison of network performance with the addition of different Shannon entropies in the descriptor set. IC_50_ values of tissue factor pathway inhibitor were predicted and analyzed using MAPE, RMSE and R^2^ of fit metrics. The descriptor set containing MW, Shannon (SMILES), Shannon (SMARTS) and Shannon (InChiKey) showed the best performance in comparison to other descriptors (blue dash) in the triangular radar graph. (b) Cumulative enhancement of network performance using Shannon descriptors depicted in the radar graph. The target was MW normalized BEI of ligands to the tissue factor pathway inhibitor, i.e. in the form of BEI/MW. The Shannon entropy framework containing MW, Shannon (SMILES) and fractional Shannon (SMILES) showed the best comparative performance in all tested metrics. (c) Comparison of the Shannon entropy framework (SEF) to Morgan fingerprint-based descriptors showed better performance of the former in predicting MW normalized BEI under the same training and testing conditions. The network performance, in all metrics, improved further when Shannon entropies were used in combination with Morgan fingerprints as descriptors. The scaling factors of all metrics were listed in Tables S3-S5. The machine learning model was based on an MLP-based deep neural network and all prediction metrics were averaged over at least 5 independent runs.

| 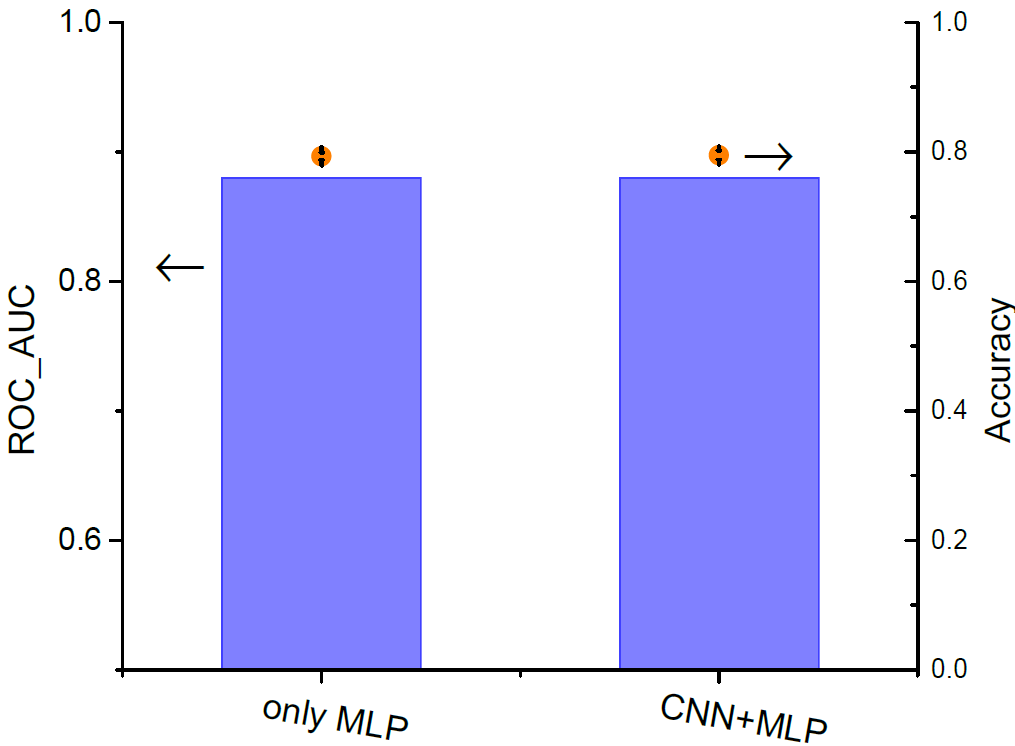  **a** |
| --- |
| 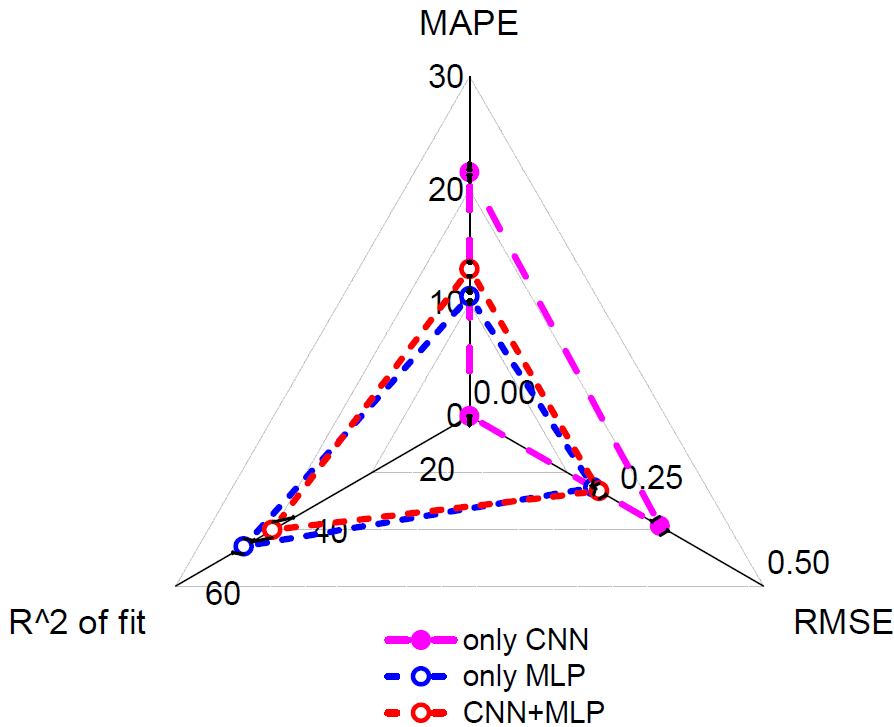  **b** |

**Fig.S3. A hybrid neural network combining MLP and CNN models shows comparable prediction performance to only MLP-based models in both classification and regression problems.** (a) The performance of only MLP and hybrid MLP+CNN models is similar as depicted by ROC_AUC and accuracy metrics in classifying toxicity data as per the Ames mutagenicity dataset. (b) Only the MLP-based network is marginally better than hybrid MLP+CNN-based network models in predicting K_i_ values of binding molecules to the coagulation factor 11 (F11). No synergy and performance gain was achieved with an MLP+CNN-based hybrid network over only an MLP-based network. The scaling factors of all metrics were listed in Table S9. All prediction metrics were averaged over at least 5 independent runs.


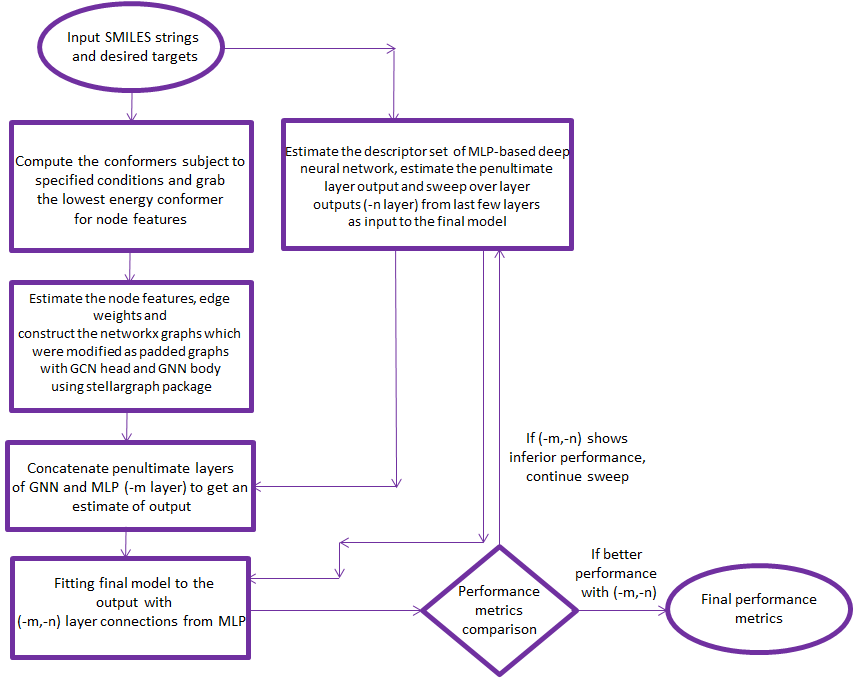


**Fig.S4. Schematic with a stepwise depiction of the used algorithm of the hybrid MLP + 3D GNN-based model to predict the logP values of binding molecules to the p53-binding protein Mdm2.**

| 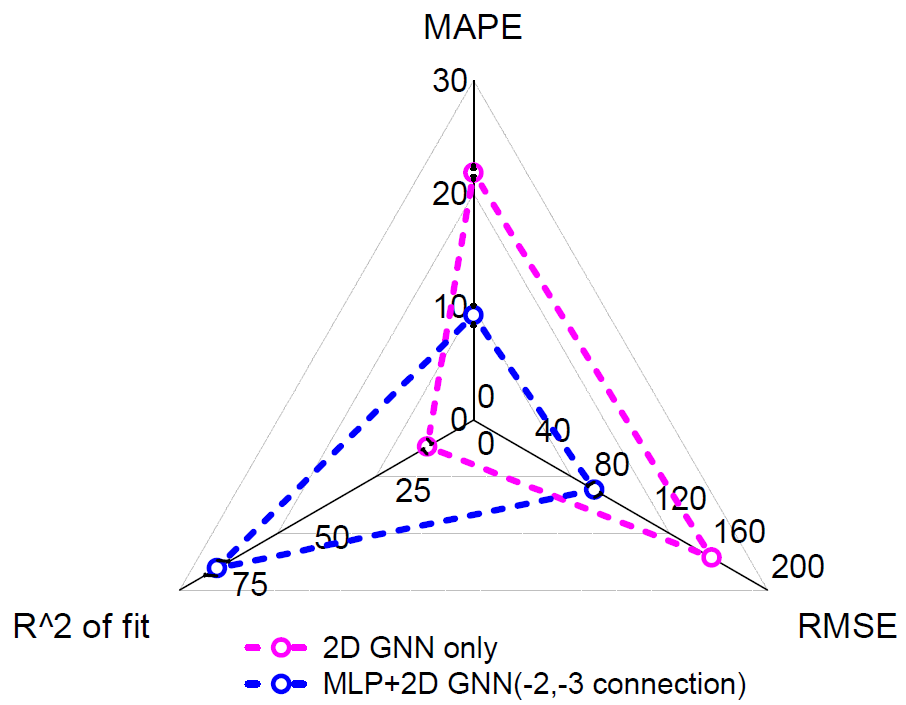  **a** |
| --- |
| 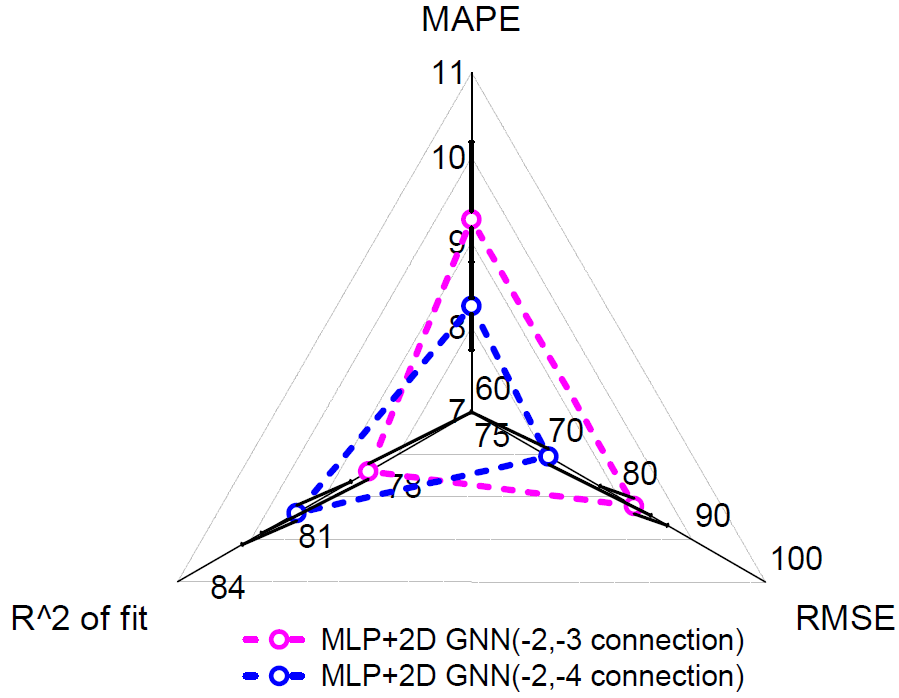  **b** |

**Fig.S5. Deep neural network model with MLP and 2D GNN architectures in an ensemble performs better than only the 2D GNN-based model and the prediction accuracy depends on the connections from MLP layers to the final, hybrid model.** (a) Prediction of logP values of binding molecules to the p53-binding protein Mdm2 is significantly more accurate when an ensemble of MLP and 2D GNN models are used than just the 2D GNN-based network. (b) Prediction performance was further enhanced by using (-2,-4) layer connections from MLP than using (-2,-3) layer connections from MLP to the final model. ‘-2’ refers to the MLP layer whose output was used to evaluate a prediction estimate which was used to fit the final model and ‘-4’ refers to the MLP layer whose output was directly used as an input to the final model. The scaling factors of all metrics were listed in Table S10.

| 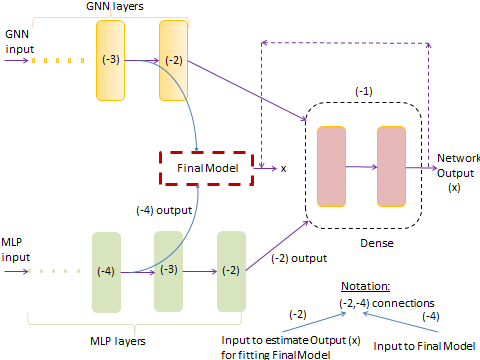  **a** |
| --- |
| 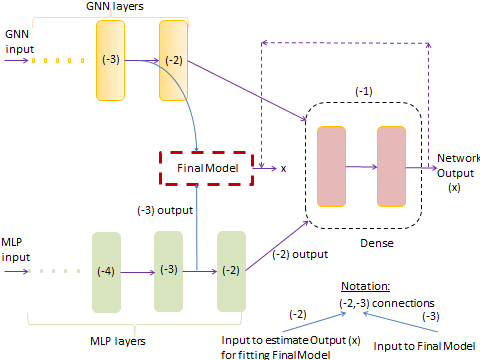  **b**  **b** |

**Fig.S6. Schematic presentations of (-2,-4) and (-2,-3) connections from the last few MLP layers to the final model of the hybrid MLP + 2D/ 3D GNN-based deep neural network.** (a) (-2,-4) connections from the MLP branch to the final model and (b) (-2,-3) connections from the MLP branch to the final model.

Table S1a. An example of numerical reduction of a molecule in the form of Shannon entropy

| **SMILES** | **Generated Tokens** | **# of different tokens**  **(in array form)** | **Shannon (SMILES)**  **Entropy**  $\boldsymbol{-}\sum_{\boldsymbol{i}}^{\boldsymbol{N}} \boldsymbol{f}_{\boldsymbol{i}}\boldsymbol{log}_{\boldsymbol{2}}\boldsymbol{f}_{\boldsymbol{i}}$ |
| --- | --- | --- | --- |
| CCN1C(=O)CC(SC[C@H](NC(=O)[C@H](CC(C)C)NC(=O)[C@H](CCCCN)NC(=O)[C@H](C)NC(=O)C(C)(C)NC(=O)[C@H](CC(C)C)NC(=O)[C@H](CCCNC(=N)N)NC(=O)[C@H](CCC(=O)O)NC(=O)[C@H](Cc2ccccc2)NC(=O)[C@H](Cc2ccc(O)cc2)NC(=O)CNC(=O)[C@H](CC(=O)O)NC(=O)[C@H](CC(F)(F)F)NC(=O)[C@H](Cc2cnc[nH]2)NC(=O)[C@@H](NC(=O)[C@H](CC(N)=O)NC(=O)[C@H]2CCCN2C(=O)[C@H](CCCCN)NC(=O)[C@H](CO)NC(=O)[C@H](CCC(N)=O)NC(=O)[C@H](Cc2ccccc2)NC(C)=O)C(C)C)C(N)=O)C1=O | 'C', 'C', 'N', '1', 'C', '(', '=', 'O', ')', 'C', 'C', '(', 'S', 'C', '[C@H]', '(', 'N', 'C', '(', '=', 'O', ')', '[C@H]', '(', 'C', 'C', '(', 'C', ')', 'C', ')', 'N', 'C', '(', '=', 'O', ')', '[C@H]', '(', 'C', 'C', 'C', 'C', 'N', ')', 'N', 'C', '(', '=', 'O', ')', '[C@H]', '(', 'C', ')', 'N', 'C', '(', '=', 'O', ')', 'C', '(', 'C', ')', '(', 'C', ')', 'N', 'C', '(', '=', 'O', ')', '[C@H]', '(', 'C', 'C', '(', 'C', ')', 'C', ')', 'N', 'C', '(', '=', 'O', ')', '[C@H]', '(', 'C', 'C', 'C', 'N', 'C', '(', '=', 'N', ')', 'N', ')', 'N', 'C', '(', '=', 'O', ')', '[C@H]', '(', 'C', 'C', 'C', '(', '=', 'O', ')', 'O', ')', 'N', 'C', '(', '=', 'O', ')', '[C@H]', '(', 'C', 'c', '2', 'c', 'c', 'c', 'c', 'c', '2', ')', 'N', 'C', '(', '=', 'O', ')', '[C@H]', '(', 'C', 'c', '2', 'c', 'c', 'c', '(', 'O', ')', 'c', 'c', '2', ')', 'N', 'C', '(', '=', 'O', ')', 'C', 'N', 'C', '(', '=', 'O', ')', '[C@H]', '(', 'C', 'C', '(', '=', 'O', ')', 'O', ')', 'N', 'C', '(', '=', 'O', ')', '[C@H]', '(', 'C', 'C', '(', 'F', ')', '(', 'F', ')', 'F', ')', 'N', 'C', '(', '=', 'O', ')', '[C@H]', '(', 'C', 'c', '2', 'c', 'n', 'c', '[nH]', '2', ')', 'N', 'C', '(', '=', 'O', ')', '[C@@H]', '(', 'N', 'C', '(', '=', 'O', ')', '[C@H]', '(', 'C', 'C', '(', 'N', ')', '=', 'O', ')', 'N', 'C', '(', '=', 'O', ')', '[C@H]', '2', 'C', 'C', 'C', 'N', '2', 'C', '(', '=', 'O', ')', '[C@H]', '(', 'C', 'C', 'C', 'C', 'N', ')', 'N', 'C', '(', '=', 'O', ')', '[C@H]', '(', 'C', 'O', ')', 'N', 'C', '(', '=', 'O', ')', '[C@H]', '(', 'C', 'C', 'C', '(', 'N', ')', '=', 'O', ')', 'N', 'C', '(', '=', 'O', ')', '[C@H]', '(', 'C', 'c', '2', 'c', 'c', 'c', 'c', 'c', '2', ')', 'N', 'C', '(', 'C', ')', '=', 'O', ')', 'C', '(', 'C', ')', 'C', ')', 'C', '(', 'N', ')', '=', 'O', ')', 'C', '1', '=', 'O' | [‘C’,’N’, ‘1’,’ (‘, ‘=’,’o’,’)’,’S’,’[C@H]’,’c’,’2’,’F’, ‘n’,’[nH]’,’[C@@H]’] =>  [78, 30, 2, 55, 29, 32, 55, 1, 18, 21, 10, 3, 1, 1, 1]  Notations:  Frequency of i-th-token :  f_i_ = n_i_/N  n_i_: # of occurrence of i-th token  N: # of tokens | 3.11 |

Table S1b. An example of numerical reduction of a molecule in the form of fractional Shannon entropy

| **SMILES** | **Shannon (SMILES)**  **Entropy** | **Fractional**  **Shannon**  **entropy (SMILES) array of the molecule (m = 27 atoms) with 0.0 padding for N = 47 maximum number of atoms in the dataset** |
| --- | --- | --- |
| COc1nn2cc(-c3cc4c(OCc5ccccc5)cccc4o3)nc2s1 | 3.0634 | [0.0  0.0  0.0  0.0  0.0  0.0  0.0  0.0  0.0  0.0  2.2692  0.3403  2.2692  0.3403  0.3403  2.2692  2.2692  2.2692  2.2692  2.2692  2.2692  0.3403  2.2692  2.2692  2.2692  2.2692  2.2692  2.2692  2.2692  2.2692  2.2692  2.2692  2.2692  0.3403  0.3403  2.2692  0.1134  0.0  0.0  0.0  0.0  0.0  0.0  0.0  0.0  0.0  0.0] |

Table S2. Stereochemistry-sensitive numerical reduction of molecules in the form of Shannon entropies by using a combination of SMILES and InChiKey strings

| **Molecules** | **SMILES**  **(Isomeric)** | **InChiKey** | **Shannon (SMILES)** | **Shannon**  **(InChiKey)** |
| --- | --- | --- | --- | --- |
| D-Glucose | C([C@@H]1[C@H]([C@@H]([C@H](C(O1)O)O)O)O)O | WQZGKKKJIJFFOK-GASJEMHNSA-N | 2.64 | 2.60 |
| L-Glucose | C([C@@H]([C@@H]([C@H]([C@@H](C=O)O)O)O)O)O | GZCGUPFRVQAUEE-VANKVMQKSA-N | 2.55 | 2.77 |
| R-Ibuprofen | C[C@H](C1=CC=C(C=C1)CC(C)C)C(=O)O | HEFNNWSXXWATRW-SNVBAGLBSA-N | 2.41 | 3.21 |
| S-Ibuprofen | C[C@@H](C1=CC=C(C=C1)CC(C)C)C(=O)O | HEFNNWSXXWATRW-JTQLQIEISA-N | 2.41 | 2.60 |
| Note: (i) ‘H’ atom was considered while estimating Shannon entropies | | | | |

Table S3. Network performance metrics for prediction of IC_50_ values of binding molecules to tissue factor pathway inhibitor (target: pCheMBL/MW, MLP-based deep neural model)

| **Descriptor set** | **MAPE (%)** | **MAE (×10^5^)** | **RMSE**  **(×10^5^)** | **R^2^ of fit (%)** |
| --- | --- | --- | --- | --- |
| kNN | 5.88 | 14.80 | 22.60 | 77.00 |
| Only MW | 10.79±0.09 | 26.02±0.08 | 34.3±0.18 | 43.5±0.70 |
| MW + Shannon(SMILES) | 8.09±0.05 | 20.81±0.12 | 30.0±0.30 | 59.47±0.69 |
| MW+Shannon(SMARTS) | 8.05±0.06 | 20.95±0.07 | 30.2±0.73 | 59.85±0.54 |
| MW+Shannon(InChiKey) | 10.22±0.12 | 25.21±0.19 | 32.8±0.25 | 49.0±1.1 |
| MW+ Shannon (SMILES) + Shannon(SMARTS)+ Shannon (InChiKey) | 8.04±0.08 | 20.92±0.24 | 29.8±0.47 | 60.45±0.93 |
| MW+fractional Shannon(SMILES) | 8.47±0.30 | 21.70±0.58 | 31.1±0.55 | 56.77±2.43 |
| MW+Shannon (SMILES)+fractional Shannon(SMILES) | 3.69±0.09 | 9.52±0.27 | 17.7±0.58 | 85.2±1.2 |
| Note: (i) No ‘H’ atom was considered while estimating Shannon entropies,  (ii) The error bars were standard deviations over a minimum sample size of 5 | | | | |

Table S4. Network performance metrics for prediction of BEI values of binding molecules to the tissue factor pathway inhibitor (target: BEI /MW, MLP-based deep neural model)

| **Descriptor set** | **MAPE (%)** | **MAE (×10^5^)** | **RMSE**  **(×10^5^)** | **R^2^ of fit (%)** |
| --- | --- | --- | --- | --- |
| kNN | 6.54 | 6.47 | 9.84 | 83.30 |
| Only MW | 10.08±0.11 | 9.58±0.09 | 12.4±0.11 | 73.20±0.81 |
| MW + Shannon(SMILES) | 7.69±0.06 | 7.73±0.08 | 10.8±0.01 | 80.61±0.36 |
| MW+ Shannon (SMILES) + fractional Shannon(SMILES) | 3.51±0.15 | 3.52±0.09 | 6.62±0.13 | 92.30±0.32 |
| MW+Morgan fingerprint | 4.65±0.05 | 4.67±0.03 | 7.20±0.17 | 90.72±0.46 |
| MW+Morgan fingerprint+ Shannon (SMILES) + fractional Shannon(SMILES) | 2.71±0.18 | 2.69±0.19 | 5.10±0.15 | 95.34±0.39 |
| Shannon (SMILES) + fractional Shannon(SMILES)^a^ | 3.67±0.14 | 3.65±0.11 | 6.68±0.31 | 92.11±0.10 |
| Note: (i) No ‘H’ atom was considered while estimating Shannon entropies  (ii) The error bars were standard deviations over a minimum sample size of 5  (iii) ^a^ case: > 5 independent runs were carried out | | | | |

Table S5. Network performance metrics for prediction of IC_50_ values of binding molecules to tissue factor pathway inhibitor in tandem approach (target: pCheMBL/MW, MLP-based deep neural model)

| **Descriptor set** | **MAPE (%)** | **MAE (×10^5^)** | **RMSE**  **(×10^5^)** | **R^2^ of fit (%)** |
| --- | --- | --- | --- | --- |
| MW+ Shannon (SMILES) + fractional Shannon(SMILES)+BEI | 1.61±0.53 | 4.07±1.33 | 6.88±1.09 | 98.20±0.51 |
| MW+BEI | 0.16±0.02 | 0.38±0.04 | 0.46±0.05 | 99.00±0.00 |
| MW+Shannon(SMILES)+ +BEI | 0.13±0.01 | 0.33±0.02 | 0.40±0.02 | 99.00±0.00 |
| Note: (i) Tandem approach refers to the prediction of BEI in the first step and then using it in the next step prediction of the IC_50_ values  (ii) No ‘H’ atom was considered while estimating Shannon entropies  (iii) The error bars were standard deviations over a minimum sample size of 5 | | | | |

Table S6. Network performance metrics for prediction of K_i_ values of binding molecules to coagulation factor 11 (target: pCheMBL/MW, MLP-based deep neural model)

| **Descriptor set** | **MAPE (%)** | **MAE**  **(×10^3^)** | **RMSE**  **(×10^4^)** | **R^2^ of fit (%)** |
| --- | --- | --- | --- | --- |
| kNN | 10.42 | 1.2 | 18.1 | 60.14 |
| MW+Shannon (SMILES) | 15.46±0.07 | 2.01±0.01 | 25.7±0.21 | 31.82±1.14 |
| MW+fractional Shannon(SMILES | 11.25±0.65 | 1.47±0.06 | 23.10±1.9 | 40.72±4.65 |
| MW+ Shannon (SMILES)+fractional Shannon(SMILES) | 10.57±0.25 | 1.41±0.06 | 23.40±0.80 | 42.91±2.23 |
| MW+ Shannon (SMILES) + Shannon(SMARTS)+ Shannon (InChiKey) )+fractional Shannon(SMILES) | 10.76±0.08 | 1.41±0.01 | 21.10±0.31 | 52.24±2.00 |
| MW+ Shannon (SMILES) + Shannon(SMARTS)+ Shannon (InChiKey) )+fractional Shannon(SMILES)+bond freq | 10.52±0.56 | 1.36±0.05 | 19.6±1.18 | 58.10±5.06 |
| Note: (i) No ‘H’ atom was considered while estimating Shannon entropies  (ii) The error bars were standard deviations over a minimum sample size of 5  (iii) The bond frequency estimates were based on the following types of bond occurrences: ['SINGLE', 'DOUBLE', 'TRIPLE', 'QUADRUPLE', 'AROMATIC', 'HYDROGEN', 'IONIC']. The output was an array containing the frequency of each bond | | | | |

Table S7. Network performance metrics for prediction of K_i_ values of binding molecules to coagulation factor 11 (target: pCheMBL/MW, MLP-based deep neural models)

| **Descriptor set** | **MAPE (%)** | **MAE (×10^3^)** | **RMSE**  **(×10^4^)** | **R^2^ of fit (%)** |
| --- | --- | --- | --- | --- |
| MW + ligands BEI | 1.95±0.18 | 0.23±0.02 | 5.67±0.35 | 96.73±0.55 |
| MW + ligands BEI+Shannon(SMILES) | 1.81±0.14 | 0.21±0.02 | 4.39±0.10 | 98.21±0.18 |

Table S8. Network performance metrics for toxicity classification as per Ames mutagenicity (target: toxicity binary classification, MLP-based & CNN&MLP –based deep neural models)

| **Descriptor set** | **ROC_AUC** | **Accuracy** |
| --- | --- | --- |
| kNN | 0.77 | 0.78 |
| MW+Shannon (SMILES) | 0.59±0.01 | 0.57±0.00 |
| MW+fractional Shannon(SMILES) | 0.7±0.00 | 0.65±0.01 |
| MW+ fractional Shannon (InChiKey) | 0.73±0.00 | 0.65±0.01 |
| MW+ Shannon (SMILES) + Shannon (SMARTS) + Shannon (InChiKey) + fractional Shannon (InChiKey) + bond freq | 0.74±0.00 | 0.65±0.01 |
| MW+ Other descriptors + Shannon (SMILES) + fractional Shannon (SMILES) | 0.88±0.00 | 0.80±0.00 |
| CNN (2-dimensional images) + MLP with descriptors: MW+ Other descriptors + Shannon (SMILES) + fractional Shannon (SMILES) | 0.88±0.00 | 0.80±0.01 |
| Note: The ‘Other descriptors’ in MLP consist of the following and all were estimated using rdkit package: molecular weight, logP, # of hydrogen bond donor, # of h bond acceptors, # of rotatable bonds, # of atoms, molar refractivity, topological surface area mapping, formal charge, # of heavy atoms, # of rings, all Lipinski module descriptors, QED properties, molecular fingerprinting with MHFP encoder and projection of Lipinski module (functional space) to MHFP encoder (structural space) | | |

Table S9. Network performance metrics for prediction of K_i_ values of binding molecules to coagulation factor 11 (target: pCheMBL/MW, MLP-based and CNN&MLP –based deep neural models)

| **Model** | **MAPE (%)** | **MAE (×10^3^)** | **RMSE**  **(×10^4^)** | **R^2^ of fit (%)** |
| --- | --- | --- | --- | --- |
| CNN | 21.52±0.23 | 2.62±0.10 | 32.40±1.40 | 0.24±0.17 |
| MLP | 10.76±0.10 | 1.42±0.02 | 23.40±0.80 | 52.24±2.01 |
| MLP+CNN | 12.98±0.59 | 1.63±0.05 | 22.10±1.15 | 46.85±3.94 |

Table S10. Network performance metrics for prediction of partition coefficient (logP) values of binding molecules to the p53-binding protein Mdm2 (target: logP) and IC_50_ (pCheMBL) values of target ID CHEMBL4691

| **Model** | **Model type** | **Descriptor set** | **MAPE (%)** | **MAE** | **RMSE**  **(×10^2^)** | **R^2^ of fit (%)** |
| --- | --- | --- | --- | --- | --- | --- |
| KNN | baseline  (logP) | n/a | 8.33 | 0.50 | 78.00 | 79.00 |
| MLP | n/a | MW | 12.82±0.47 | 0.85±0.03 | 142.44±4.60 | 33.72±4.43 |
|  |  | MW + fractional Shannon(SMILES) | 11.35±1.60 | 0.54±0.03 | 87.85±9.08 | 73.78±4.58 |
|  |  | MW+Shannon(SMILES)+fractional Shannon(SMILES) | 9.45±1.25 | 0.49±0.02 | 80.35±5.90 | 78.45±2.58 |
| GNN | 2D GNN | node features: atomic mass + fractional Shannon(SMILES) | 23.5±0.05 | 1.08±0.01 | 175.02±0.85 | 0.23±0.03 |
|  | 3D GNN | node features: atomic mass + fractional Shannon(SMILES) | 10.43±0.21 | 0.66±0.02 | 100.74±1.26 | 69.60±1.73 |
|  | 3D  GNN | node features: atomic mass + fractional Shannon(SMILES)+Shannon(SMILES) | 9.39±0.19 | 0.60±0.03 | 94.71±5.41 | 72.60±1.30 |
| MLP  +  GNN | 3D GNN + MLP  (-2,-2)  connection | node features: atomic mass + fractional Shannon(SMILES)+Shannon(SMILES)  MLP descriptors: MW+ fractional Shannon(SMILES)+Shannon(SMILES) | 9.67±0.23 | 0.56±0.03 | 77.0±5.19 | 79.8±2.8 |
|  | 3D GNN + MLP  (-2,-3)  connection | node features: atomic mass + fractional Shannon(SMILES)+Shannon(SMILES)  MLP descriptors: MW+ fractional Shannon(SMILES)+Shannon(SMILES) | 8.44±0.48 | 0.51±0.04 | 79.10±8.40 | 78.62±4.86 |
|  | 3D  GNN + MLP  (-2,-4)  connection | node features: atomic mass + fractional Shannon(SMILES)+Shannon(SMILES)  MLP descriptors: MW+ fractional Shannon(SMILES) | 8.15±0.56 | 0.48±0.04 | 72.43±6.37 | 82.07±3.44 |
|  | 2D GNN + MLP  (-2,-2)  connection | node features: atomic mass + fractional Shannon(SMILES)+Shannon(SMILES)  MLP descriptors: MW+ fractional Shannon(SMILES) | 9.53±0.26 | 0.56±0.03 | 77.76±5.20 | 79.00±3.41 |
|  | 2D GNN + MLP  (-2,-3)  connection | node features: atomic mass + fractional Shannon(SMILES)+Shannon(SMILES)  MLP descriptors: MW+ fractional Shannon(SMILES) | 9.27±0.92 | 0.55±0.05 | 82.11±4.60 | 78.50±3.64 |
|  | 2D GNN + MLP  (-2,-4)  connection | node features: atomic mass + fractional Shannon(SMILES)+Shannon(SMILES)  MLP descriptors: MW+ fractional Shannon(SMILES) | 8.25±0.52 | 0.51±0.03 | 70.50±13.90 | 81.00±1.85 |
| KNN^a^ | baseline (pChEMBL) | n/a | 13.70 | 2.25 | 2.90 | 63.84 |
| MLP  +  GNN^a^ | 3D  GNN + MLP  (-2,-4)  connection | node features: atomic mass + fractional Shannon(SMILES)+Shannon(SMILES)  MLP descriptors: MW+ fractional Shannon(SMILES) | 11.58±0.26 | 1.91±0.07 | 2.45±0.06 | 72.56±1.20 |
| Note: (i) The error bars were standard deviations over a minimum sample size of 5,  (ii) ^a^ The target ID was CHEMBL4691 and the target was IC_50_ in pChEMBL format. The scaling factor for both MAE and RMSE was 10^3^ | | | | | | |

Table S11. Network performance metrics for prediction of BEI and pChEMBL values across different target datasets using MLP-based deep neural network architecture

| **Target**  **ID** | **Descriptor type** | **MAPE (%)** | **MAE** | **RMSE** | **R^2^ of fit (%)** |
| --- | --- | --- | --- | --- | --- |
| CHEMBL  3713062^a^  (BEI) | Morgan | 4.95±1.27 | 5.00±0.13 | 7.50±0.26 | 90.00±0.70 |
|  | SEF | 3.71±0.15 | 3.70±0.15 | 6.84±0.45 | 91.70±1.17 |
|  | SHED | 10.55±0.30 | 10.74±0.48 | 15.45±0.88 | 61.37±3.03 |
|  | kNN | 6.54 | 6.47 | 9.84 | 83.30 |
| CHEMBL  204  (BEI) | Morgan | 17.42±0.49 | 5.03±0.18 | 11.56±0.75 | 62.18±0.73 |
|  | SEF | 15.10±0.16 | 4.23±0.04 | 7.65±0.31 | 85.66±0.95 |
|  | SHED | 35.57±0.78 | 10.41±0.30 | 18.35±0.42 | 57.68±0.56 |
|  | kNN | 15.05 | 4.20 | 9.38 | 71.6 |
| CHEMBL  274  (BEI) | Morgan | 12.86±0.92 | 3.54±0.24 | 5.24±0.18 | 67.50±1.61 |
|  | SEF | 10.38±0.28 | 2.90±0.05 | 4.57±0.27 | 75.86±3.08 |
|  | SHED | 17.57±0.08 | 4.95±0.02 | 7.20±0.06 | 37.67±1.27 |
|  | kNN | 10.31 | 2.64 | 4.14 | 78.40 |
| CHEMBL  2842  (BEI) | Morgan | 12.39±0.24 | 4.50±0.14 | 6.31±0.44 | 82.02±1.92 |
|  | SEF | 11.28±0.18 | 4.07±0.08 | 5.83±0.24 | 84.60±0.91 |
|  | SHED | 27.12±1.29 | 9.76±0.24 | 13.38±0.59 | 17.63±2.17 |
|  | kNN | 14.26 | 4.90 | 7.13 | 76.6 |
| CHEMBL  3974  (BEI) | Morgan | 15.16±0.77 | 5.00±0.35 | 6.82±0.48 | 84.83±1.73 |
|  | SEF | 10.68±0.29 | 3.52±0.11 | 4.94±0.18 | 91.65±0.55 |
|  | SHED | 27.58±0.02 | 9.23±0.08 | 12.06±0.11 | 58.35±0.24 |
|  | kNN | 12.33 | 3.79 | 5.63 | 74.56 |
| CHEMBL  2820  (BEI) | Morgan | 16.65±1.05 | 3.33±0.25 | 4.32±0.26 | 64.00±3.47 |
|  | SEF | 13.42±0.76 | 2.92±0.11 | 3.88±0.20 | 74.38±1.51 |
|  | SHED | 23.58±1.01 | 4.58±0.13 | 5.63±0.16 | 42.50±0.51 |
|  | kNN | 12.64 | 2.70 | 3.70 | 74.34 |
| CHEMBL  2815  (BEI) | Morgan | 12.03±0.48 | 4.21±0.21 | 6.20±0.33 | 70.83±2.90 |
|  | SEF | 10.70±0.11 | 3.84±0.07 | 5.63±0.15 | 78.80±0.76 |
|  | SHED | 19.01±0.02 | 7.25±0.02 | 10.91±0.02 | 21.61±0.08 |
|  | kNN | 11.40 | 3.90 | 5.80 | 74.30 |
| CHEMBL  4691  (pChEMBL) | Morgan | 12.45±0.27 | 2.14±0.08 | 2.70±0.07 | 67.00±1.92 |
|  | SEF | 11.47±0.29 | 1.94±0.03 | 2.52±0.03 | 72.00±1.31 |
|  | SHED | 15.84±0.03 | 2.70±0.02 | 3.39±0.04 | 49.44±0.11 |
|  | kNN | 13.70 | 2.25 | 2.90 | 63.84 |
| Note: (i) ^a^The scaling factor in MAE was 10^5^ and for the rest of the Target IDs the scaling factor was 10^3^  (ii) all y-values were normalized with respective MWs during training/ testing  (iii) scikit-learn train_test_split function was used to split the dataset at train : test = 80% : 20% ratio with shuffle ~ True and random_state ~ 42 | | | | | |

Table S12. Comparison of Morgan, SEF, SHED, SEF+Morgan and SEF+SHED descriptors in random forest regression-based models

| **Target**  **ID** | **Descriptor type** | **MAPE (%)** | **MAE** | **RMSE** | **R^2^ of fit (%)** |
| --- | --- | --- | --- | --- | --- |
| CHEMBL  3713062^a^  (BEI) | kNN | 6.54 | 6.47 | 9.84 | 83.30 |
|  | Morgan | 4.86±0.01 | 4.75±0.01 | 7.04±0.07 | 91.10±0.20 |
|  | SEF | 3.00±0.03 | 2.92±0.03 | 5.38±0.04 | 95.00±0.07 |
|  | SHED | 4.7±0.01 | 4.51±0.02 | 7.46±0.03 | 90.10±0.10 |
|  | SEF+Morgan | 2.47±0.03 | 2.36±0.02 | 4.00±0.02 | 97.2±0.04 |
|  | SEF+SHED | 3.00±0.04 | 2.92±0.03 | 5.45±0.02 | 94.80±0.01 |
| CHEMBL  204  (BEI) | kNN | 15.05 | 4.20 | 9.38 | 71.60 |
|  | Morgan | 19.47±0.31 | 4.60±0.05 | 9.55±0.1 | 71.70±0.70 |
|  | SEF | 14.30±0.10 | 3.65±0.03 | 6.10±0.09 | 88.30±0.36 |
|  | SHED | 28.60±0.20 | 7.10±0.05 | 12.92±0.21 | 46.70±1.87 |
|  | SEF+Morgan | 12.32±0.03 | 3.23±0.00 | 6.05±0.00 | 89.00±0.04 |
|  | SEF+SHED | 13.84±0.04 | 3.60±0.00 | 6.20±0.03 | 88.0±0.15 |
| CHEMBL  274  (BEI) | kNN | 10.31 | 2.64 | 4.14 | 78.40 |
|  | Morgan | 8.60±0.15 | 2.2±0.04 | 3.30±0.05 | 85.30±0.54 |
|  | SEF | 8.23±0.04 | 2.09±0.01 | 3.20±0.03 | 86.00±0.26 |
|  | SHED | 14.90±0.10 | 3.85±0.01 | 5.86±0.06 | 54.74±0.50 |
|  | SEF+Morgan | 7.23±0.01 | 1.85±0.00 | 2.80±0.02 | 89.20±0.20 |
|  | SEF+SHED | 8.25±0.06 | 2.10±0.00 | 3.18±0.01 | 86.000.±.10 |
| CHEMBL  2842  (BEI) | kNN | 14.26 | 4.90 | 7.13 | 76.6 |
|  | Morgan | 11.9±0.03 | 4.10±0.02 | 6.20±0.00 | 82.30±0.05 |
|  | SEF | 9.80±0.02 | 3.61±0.00 | 5.27±0.00 | 87.00±0.01 |
|  | SHED | 23.26±0.20 | 8.35±0.05 | 11.66±0.08 | 37.00±0.80 |
|  | SEF+Morgan | 8.46±0.01 | 3.13±0.01 | 4.67±0.00 | 90.00±0.03 |
|  | SEF+SHED | 9.77±0.14 | 3.60±0.01 | 5.25±0.02 | 87.00±0.13 |
| CHEMBL  5023^b^  (logP) | kNN | 8.33 | 0.50 | 0.78 | 79.00 |
|  | Morgan | 8.33±0.12 | 0.51±0.00 | 0.77±0.01 | 80.15±0.43 |
|  | SEF | 7.10±0.22 | 0.43±0.01 | 0.65±0.01 | 87.00±0.37 |
|  | SHED | 12.64±0.45 | 0.63±0.01 | 0.94±0.02 | 72.00±1.10 |
|  | SEF+Morgan | 7.23±0.12 | 0.43±0.00 | 0.69±0.01 | 84.60±0.51 |
|  | SEF+SHED | 7.00±0.04 | 0.40±0.00 | 0.61±0.01 | 88.50±0.30 |
| Note:(i) ^a^The scaling factor in MAE was 10^5^, ^b^the scaling factor was 1 and for the rest of the Target IDs the scaling factor was 10^3^  (ii) all y-values were normalized with respective MWs during training/ testing, except CHEMBL5023  (iii) scikit-learn train_test_split function was used to split the dataset at train : test = 80% : 20% ratio with shuffle ~ True and random_state ~ 42; ^b^ dataset split at train : test = 85% : 15% ratio with shuffle ~ True | | | | | |

Table S13. Features used in constructing SEF descriptors for optimum performance in random forest regression-based models

| **Target**  **ID** | **SEF features** |
| --- | --- |
| CHEMBL  3713062  (BEI) | fractional Shannon (SMILES) + Shannon entropy of bonds |
| CHEMBL  204  (BEI) | fractional Shannon (SMILES) + Shannon entropy of bonds |
| CHEMBL  274  (BEI) | fractional Shannon (SMILES) + bond frequency + Shannon entropy (InChiKey) |
| CHEMBL  2842  (BEI) | fractional Shannon (SMILES) + bond frequency + Shannon entropy (SMILES) |
| CHEMBL  5023  (LogP) | fractional Shannon (SMILES) + bond frequency |

Table S14. Comparison of Morgan, SEF and SHED descriptors in random forest regression-based models with completely random y-label targets

| **Target**  **ID** | **Descriptor type** | **MAPE (%)** | **MAE** | **RMSE** | **R^2^ of fit (%)** |
| --- | --- | --- | --- | --- | --- |
| CHEMBL  3713062^a^  (BEI) | Morgan | 5.05±0.34 | 4.83±0.28 | 7.40±0.44 | 90.32±1.70 |
|  | SEF | 3.22±0.14 | 3.09±0.24 | 5.78±0.41 | 94.46±0.85 |
|  | SHED | 4.88±0.02 | 4.63±0.17 | 8.44±0.76 | 87.41±2.53 |
|  | SEF+Morgan | 2.51±0.20 | 2.34±0.10 | 4.14±0.25 | 97.10±0.40 |
|  | SEF+SHED | 2.85±0.10 | 2.73±0.13 | 5.10±0.44 | 95.55±0.62 |
| CHEMBL  204  (BEI) | Morgan | 18.20±1.75 | 4.62±0.38 | 10.92±3.06 | 71.84±10.57 |
|  | SEF | 13.11±0.62 | 3.54±0.28 | 6.44±1.25 | 86.10±3.75 |
|  | SHED | 29.67±1.91 | 7.75±0.43 | 14.60±2.02 | 42.42±9.83 |
| CHEMBL  2842  (BEI) | Morgan | 11.96±0.20 | 3.94±0.13 | 5.90±0.14 | 82.42±1.38 |
|  | SEF | 9.10±0.34 | 3.21±0.15 | 4.66±0.25 | 89.40±1.60 |
|  | SHED | 23.35±0.56 | 8.00±0.29 | 11.27±0.42 | 38.55±3.40 |
| Note:(i) ^a^The scaling factor in MAE was 10^5^ and for the rest of the Target IDs the scaling factor was 10^3^  (ii) all y-values were normalized with respective MWs during training/ testing  (iii) scikit-learn train_test_split function was used to split the dataset at train : test = 80% : 20% ratio with shuffle ~ True | | | | | |
